# Metastatic tumor evolution and organoid modeling implicate *TGFBR2* as a cancer driver in diffuse gastric cancer

**DOI:** 10.1186/s13059-014-0428-9

**Published:** 2014-08-27

**Authors:** Lincoln D Nadauld, Sarah Garcia, Georges Natsoulis, John M Bell, Laura Miotke, Erik S Hopmans, Hua Xu, Reetesh K Pai, Curt Palm, John F Regan, Hao Chen, Patrick Flaherty, Akifumi Ootani, Nancy R Zhang, James M Ford, Calvin J Kuo, Hanlee P Ji

**Affiliations:** Division of Oncology, Department of Medicine, Stanford University School of Medicine, CCSR 1115, 269 Campus Drive, Stanford, CA 94305-5151 USA; Stanford Genome Technology Center, Stanford University, Palo Alto, CA 94304 USA; Department of Pathology, University of Pittsburgh Medical Center, Pittsburgh, PA 15213 USA; Bio-Rad, Inc, Pleasanton, CA 94566 USA; Department of Statistics, Stanford University, Stanford, CA 94305 USA; Division of Hematology, Department of Medicine, Stanford University School of Medicine, CCSR 1155, 269 Campus Drive, Stanford, CA 94305-5151 USA; Department of Statistics, The Wharton School, University of Pennsylvania, Philadelphia, PA 19104 USA

## Abstract

**Background:**

Gastric cancer is the second-leading cause of global cancer deaths, with metastatic disease representing the primary cause of mortality. To identify candidate drivers involved in oncogenesis and tumor evolution, we conduct an extensive genome sequencing analysis of metastatic progression in a diffuse gastric cancer. This involves a comparison between a primary tumor from a hereditary diffuse gastric cancer syndrome proband and its recurrence as an ovarian metastasis.

**Results:**

Both the primary tumor and ovarian metastasis have common biallelic loss-of-function of both the *CDH1* and *TP53* tumor suppressors, indicating a common genetic origin. While the primary tumor exhibits amplification of the Fibroblast growth factor receptor 2 (*FGFR2*) gene, the metastasis notably lacks *FGFR2* amplification but rather possesses unique biallelic alterations of Transforming growth factor-beta receptor 2 (*TGFBR2*), indicating the divergent *in vivo* evolution of a *TGFBR2*-mutant metastatic clonal population in this patient. As *TGFBR2* mutations have not previously been functionally validated in gastric cancer, we modeled the metastatic potential of TGFBR2 loss in a murine three-dimensional primary gastric organoid culture. The *Tgfbr2* shRNA knockdown within *Cdh1*^*-/-*^*; Tp53*^*-/-*^ organoids generates invasion *in vitro* and robust metastatic tumorigenicity *in vivo*, confirming *Tgfbr2* metastasis suppressor activity.

**Conclusions:**

We document the metastatic differentiation and genetic heterogeneity of diffuse gastric cancer and reveal the potential metastatic role of *TGFBR2* loss-of-function. In support of this study, we apply a murine primary organoid culture method capable of recapitulating *in vivo* metastatic gastric cancer. Overall, we describe an integrated approach to identify and functionally validate putative cancer drivers involved in metastasis.

**Electronic supplementary material:**

The online version of this article (doi:10.1186/s13059-014-0428-9) contains supplementary material, which is available to authorized users.

## Background

Worldwide, gastric adenocarcinoma is the fourth most common malignancy and the second leading cause of cancer deaths among men and women. Based on distinctive histopathologic features, gastric adenocarcinoma is categorized into diffuse and intestinal subtypes [1]. In terms of histopathology, diffuse gastric cancers are generally undifferentiated, frequently have signet cell ring features and invasively infiltrate normal stomach tissue. In contrast, the intestinal subtype has epithelial features and forms discrete tumor masses similar to colon cancer. Diffuse gastric cancer has a higher incidence of metastatic disease and a generally worse prognosis compared to the intestinal subtype [2,3]. Currently, the genomic analyses of diffuse gastric cancer have involved a small number of samples including a recent study by the Cancer Genome Atlas Project (TCGA) and a whole genome sequencing survey of a set of diffuse gastric tumors [4]. However, there are few, if any, studies that detail the metastatic evolution of gastric cancer; metastatic tumors are typically absent from large-scale genomic cancer surveys such as TCGA. Overall, little is known about the oncogenic process and tumor evolution of metastatic gastric cancer despite its paramount clinical importance [5].

In hereditary diffuse gastric cancer (HDGC), germline mutations in *CDH1* (that is, E-cadherin) confer a 70% lifetime risk of developing diffuse gastric cancer [6,7]. The *CDH1* tumor suppressor gene encodes E-cadherin, a transmembrane glycoprotein that mediates calcium-dependent cell-cell adhesion. Changes in CDH1 function affect the epithelial-mesenchymal transition (EMT) that has been implicated as playing a role in tumorigenesis. Studies of affected HDGC individuals’ tumors provide a unique opportunity to determine the essential drivers of diffuse gastric cancer in the context of *CDH1* loss of function. Supporting evidence of the role of *CDH1* in sporadic diffuse gastric cancers includes the observation that 50% contain *CDH1* mutations or hypermethylation of the *CDH1* promoter [8,9]. A recent whole genome sequencing survey of diffuse gastric cancer also identified frequent *CDH1* mutations as the most common driver event [4]. The TCGA gastric cancer data also show a high frequency of somatic *CDH1* mutations [10]. Significantly less is known about the identity and role of co-occurring drivers that contribute to diffuse gastric metastasis.

**Figure 1:**
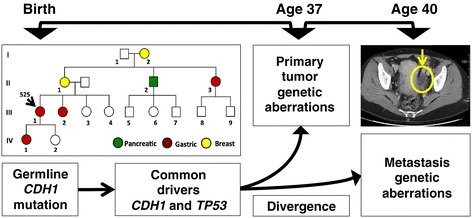
**Family and clinical history of a Mendelian diffuse gastric cancer.** The pedigree of the index patient 525 (III-1) is depicted. Tumor types are indicated by color including green for pancreatic cancer, red for diffuse gastric cancer, and yellow for breast cancer. The patient presented with her primary gastric cancer at the age of 37 years. Three years later she presented with an abdominal discomfort. Contrast-enhanced CT scan of the pelvis identified a left ovary mass (yellow circle) that was confirmed on biopsy to be a diffuse gastric cancer metastasis (that is, Krukenberg tumor). During the course of metastatic tumor evolution, a number of known and candidate cancer driver events delineated the tumor evolution and genetic divergence of the metastasis from the primary tumor.

Herein, we report a study of the metastatic evolutionary process in diffuse gastric cancer. Our goal was to identify known and candidate drivers that delineate the tumor progression during metastasis. We performed an extensive genome sequencing analysis of a primary gastric tumor and metastasis from an individual with a germline *CDH1* mutation (Figure 1) who presented with a gastric primary, followed after 3 years by metastasis in the left ovary. Given the existing germline mutation in *CDH1,* the cancer genome only requires a second allelic hit via a somatic genetic aberration, as is demonstrated in the tumor from this individual. Because the initial cancer driver event is known, Mendelian cancer genomes provide a rare and highly informative ‘experiment of nature’ that provides an opportunity to delineate somatic genetics of metastasis. Genome sequencing analysis of both tumors revealed evidence of a common origin based on shared mutations but greater genomic diversity seen both at the level of mutations as well as extensive allelic imbalance and copy number aberrations for the metatasis.

We determined if the candidate drivers from this metastatic progression were sufficient to reproduce diffuse gastric cancer. Our cancer modeling methodology used *in vitro* gastric organoids and allows one to engineer the genetic driver context of these cancers and study the process of metastatic evolution and oncogenic pathway divergence. Integrating genetic analysis and biological modeling, we determined the independent role of *TGFBR2* (transforming growth factor-β receptor 2) in the oncogenesis of diffuse gastric cancer. Our experimental cancer modeling relies on an air-liquid interface for primary mouse intestinal culture that contains both epithelial and mesenchymal elements, accurately recapitulates long-term proliferation, multilineage differentiation, the Wnt/Notch-dependent stem cell niche, and peristalsis [11]. We reported an analogous primary gastric organoid culture system that accurately recapitulates multilineage epithelial differentiation and stromal elements [12]. Recently, we achieved robust *in vitro* oncogenic transformation of primary gastric, colon, and pancreatic organoids via mutations in *Kras* and *Trp53*, which induce high-grade dysplasia and invasion *in vitro* with adenocarcinoma upon subcutaneous transplantation into mice [13]. We demonstrate the functional validation of candidate gastric cancer metastasis drivers from cancer genomic profiling studies, focusing on modeling the *TGFBR2* driver as proof of principle.

## Results

### Diffuse gastric cancer and metastatic progression

At the age of 37 years, the index patient (525) was diagnosed with stage III (T3N1M0) poorly differentiated diffuse gastric adenocarcinoma (Figure 1). Her 42-year-old sister was diagnosed with diffuse gastric adenocarcinoma 2 months earlier. Based on the family history of gastric cancer and the unusually young age of onset, she underwent germline *CDH1* mutation testing. The patient and her sister were found to have a germline splice site mutation in intron 10 (c.1565 + 2insT). This germline mutation was subsequently reported in another family with hereditary diffuse gastric cancer (HDGC) [14]. The patient underwent a total gastrectomy to remove her primary tumor and was found to have a single lymph node metastasis. She received standard adjuvant treatment including combined chemotherapy (cisplatinum and 5-fluorouracil) and radiation. Three years after her initial presentation, the patient reported progressive lower abdominal fullness. A computed tomography (CT) scan demonstrated a large pelvic mass consistent with a left ovarian metastasis (Figure 1). Subsequently, the patient underwent laparotomy with bilateral salpingo-oophorectomy and biopsy of the pelvic mass. Pathological studies demonstrated metastatic adenocarcinoma involving the ovary, otherwise referred to as a Krukenberg tumor, with the same histologic appearance as the primary tumor. One study reported that among diffuse gastric cancer with metastatic dissemination, the ovary was a metastatic site in 28.8% of cases [15]. Thus, the ovary is a common site for metastatic disease.

### Cancer genome sequencing analysis

Both exome and whole genome paired-end sequencing were performed on the primary tumor, ovarian metastasis, and normal tissue which included blood and normal gastric tissue (Additional file 1: Table S1). Tissue from the lymph node metastasis was not available for analysis. Multiple sequencing methods were employed to compensate for the extent of normal stromal mixture, a direct result of the infiltrative invasiveness of the diffuse gastric cancer subtype. We determined the extent of normal genome mixture and corrected for inclusion of the normal DNA (Additional file 1: Methods). Given the complexity of the tumor samples, we conducted an additional round of targeted sequencing to confirm the presence of mutations and other genetic aberrations that occurred in exons, near exon boundaries or promoters.

Overall, we obtained greater than 100× average coverage for each exome and generally relied on exome data for the discovery of coding region mutations. For whole genome sequencing, we had greater than 60× average coverage for the primary cancer whole genome sample and 30× for the metastatic genome. The whole genome sequencing was used for identifying larger scale genetic aberrations such as copy number variation (CNVs), allelic imbalances, rearrangements, and other classes of structural rearrangements. After alignment, we conducted variant calling to identify somatic mutations and other classes of genetic aberrations. This included somatic mutations, insertion-deletions (indels), CNVs, loss-of-heterozygosity regions (LOH), and cancer rearrangements (Additional file 1: Table S3 and Table S4). As a control for single nucleotide variant calling, we genotyped the samples with Affymetrix 6.0 single nucleotide polymorphism (SNP) arrays; we compared the genotypes to the identified SNPS from the sequence data. The concordance of exome and whole genome SNP data to the array data was 99%.

### Coding region mutations and validation with deep sequencing

We identified mutations that occurred in exons and intronic mutations within 100 bases of the exon boundary and the results are summarized in Additional file 1: Table S2. As noted previously, the tumor samples had complex composition that reduced the sequence coverage of some mutations. We proceeded with an additional round of targeted sequencing to validate these mutations and determine their presence in both tumors. We designed an assay for deep targeted resequencing that covered approximately 300 bases around the specific mutation loci (Additional file 1: Table S5). The average targeted sequencing coverage for each putative mutation or loci was 278× for the normal, 251× for the primary tumor and 152× for the metastasis.

Between the two tumors, we independently validated a total of 77 mutations that occurred within or proximal to exons (Additional file 1: Methods and Table S5). Validated genetic aberrations included: (1) non-synonymous mutations, (2) synonymous mutations, (3) insertions, or (4) deletions. With the targeted sequencing data, we determined the mutation allelic frequency (MAF) between the primary tumor and metastasis for each mutation. This involves determining the fraction of a sequence read with a mutation in comparison to the reference sequence reads. We were able to identify which mutations were common or exclusive to the primary tumor versus the metastasis. Among the 77 validated mutations, the distribution was such that mutations were generally unique either to the primary tumor or metastatic site. For example, the primary tumor had eight mutations that were not present in the metastasis while the metastasis had 37 mutations not present in the primary tumor. Common to both cancers were 32 mutations.

Given the interval of three years prior to the detection of the metastasis, there is a possibility that the metastasis-specific mutations occurred independently from the primary tumor. Mutations specific to the primary tumor that were not present in the ovarian metastasis may have been the result of random genetic drift. The mutations common to both indicate a common origin but the exact timing of the differentiation between the two tumors is less clear as noted by those mutations with lower MAF. A subset of these genes had high MAF values, indicating a higher likelihood of being present in all clonal populations in the primary tumor or metastasis. As we describe later, these genes were prioritized for further experimental testing in gastric organoids.

### Mutations affecting gene function

**Figure 2:**
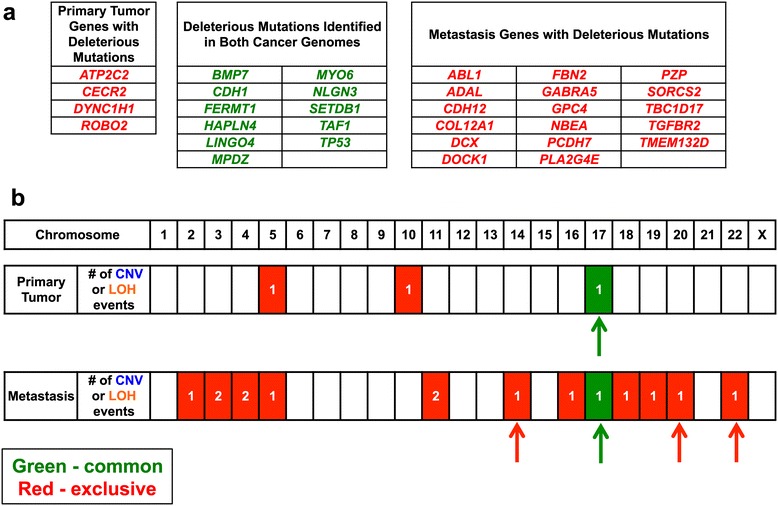
**Comparison of the genetic aberrations in the primary tumor and metastasis.** Common *versus* exclusive genetic aberrations are compared between the two tumor genomes. **(a)** Genes with coding mutations having a potential deleterious impact are listed. These genes are classified based on whether they are exclusive (red characters) or common (green characters) to the primary tumor and metastasis. The mutations all lead to changes in the amino acid composition of the gene product and were identified to have a significant alteration with a high likelihood of affecting the gene product function. **(b)** A summary of the chromosomal aberrations is shown across the entire cancer genome of both tumors. This includes copy number variation (CNV) or loss of heterozygosity (LOH). The red blocks indicates events exclusive to the primary tumor or metastasis. The green blocks indicate events common to both. The number of events per chromosome is listed in each block. Arrows indicate LOH events or deletions that encompass the p arm, q arm, or entire chromosome. Red arrows indicate chromosomal aberrations that are exclusive and green arrows indicate events that are common.

Among the mutations that were externally validated, we focused on the subset of mutations leading to amino acid substitutions, premature stop codons and indels that altered the open reading frame. Subsequently, we determined if these coding mutations were potentially deleterious to gene function using a number of prediction algorithms such as Polyphen [16] and SIFT [17] among others. Based on the MAF information for each mutation, we determined whether these mutations with a possible deleterious impact on the gene products were common or exclusive to the primary tumor and metastasis (Figure 2).

On the subset of deleterious mutations, we conducted additional biological pathway analysis, literature review and comparison against the Cancer Genome Atlas data available for diffuse gastric cancer. This identified a set of known cancer genes and likely cancer-related candidates with mutations that likely had an impact on protein function. We focused on a number of candidate driver genes (Table 1) that had previously been demonstrated to have oncogenic potential or were known tumor suppressors with biallelic changes present in the cancer genomes.

**Table 1:** **Cancer oncogenes with amplifications or cancer drivers with biallelic events**

**Origin**	**Known or candidate cancer driver**	**Biallelic event**	**Allelic alteration 1**	**Mutation or genomic aberration**	**Chr**	**Chr position or interval**	**Allelic alteration 2**
Unique to the primary	FGFR2*		Amplification	6-fold amplification	10	117820033 - 119748751	
Common to the primary tumor and metastasis	*CDH1*	Yes	Deletion	Partial deletion of exon 9	16	68847326 - 68847403	Germline mutation in *CDH1*
	*TP53*	Yes	5’ splice site mutation	Aberrant splicing	17	7578370	Hemizygous loss of 17p arm
Unique to the metastasis	*TGFBR2*	Yes	Frameshift indel	Stop codon in exon 4	3	30691871	Hemizygous deletion of wild-type *TGFBR2* locus
	*PCDH7*	Yes	Missense	S87R	4	30723305	Hemizygous deletion of wild-type 4 arm
	*FERMT1* loci	Yes	Loss of heterozygosity	*FERMT*1 located in 20p12.3	20		*FERMT*1 mutation
	*BMP7* loci	Yes	Loss of heterozygosity	*BMP7* located in 20q13.3	20		*BMP7* mutation

Chr: chromosome.

### Copy number variations and allelic imbalances distinguishing the primary tumor from the metastasis

**Figure 3:**
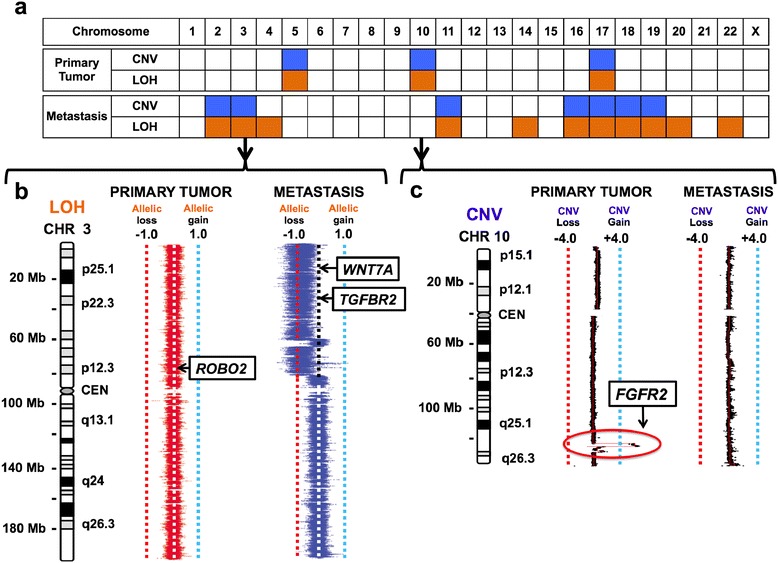
**Genetic divergence of the ovarian metastasis from the primary gastric cancer for critical candidate drivers.** The genomic position of the mutation, copy number variations (CNV) regions or loss-of-heterozygosity (LOH) intervals are shown from the cancer genomes. For the chromosome plots, the Y axis designates position with the respective chromosome, its length in megabases (MB) and ideogram designation shown to the left of the copy number profile. Deleterious mutations are shown as boxed arrows with the gene symbol. **(a)** The genome wide distribution of cancer-specific CNVs and LOH intervals are summarized across all chromosomes for the primary tumor and metastasis. **(b)** On Chromosome 3, the metastasis had unique biallelic events involving a deleterious *TGFBR2* mutation and a genomic deletion affecting the other allele as seen most clearly with LOH intervals. Secondary to genomic deletions, LOH is demonstrated as a shift in the minor allelic frequency ratio value of -1 and correlates with a genomic deletion. **(c)** On chromosome 10, the *FGFR2* gene was located in a genomic amplification region seen only in the primary and not the metastasis. The amplification is noted in a red circle.

We noted larger scale genomic aberrations that differentiated the primary from the metastasis (Figures 2b and 3a). This included copy number changes and LOH events. Unique to the primary tumor were two genomic amplifications on chromosomes 5 and 10, and two inversions on chromosomes 15 and 16 (Additional file 1: Table S4). The chromosome 10 amplification covered a 1.66 Mb interval. When considering deletions or allelic imbalances, the only major event that was noted involved a loss of the p arm of chromosome 17.

In contrast to the primary tumor, the metastatic tumor had numerous chromosomal scale LOH events and genomic deletions affecting 12 different chromosomes, the majority of which were unique to the metastatic tumor (Figure 2). This included multiple deletions and copy neutral LOH events that are detailed in Additional file 1: Table S3. There was a five-fold genomic amplification in chromosome 2 but no specific known genes existed in the affected interval. There were no detectable inter-chromosomal translocations in either the primary tumor or metastasis genomes. Other cancer rearrangements were identified but did not point towards aberrations in any candidate driver genes (Additional file 1: Table S4). There were indications of large scale genomic instability based upon allelic imbalance analysis; chromosomes 14, 17, 20, and 22 all involved the entire chromosome.

For copy number aberrations and allelic imbalances, we identified exclusive versus common events between the primary tumor and metastasis. The only common genetic aberration involved the p arm of chromosome 17. Overall, the lack of overlap was indicative of significant genetic divergence from the primary tumor and metastasis despite a common origin as denoted by shared mutations in critical tumor suppressors.

The genomic intervals of the LOH, copy number aberration and rearrangement events were compared with the position of validated gene mutations. This integrated analysis pointed to a number of genes that had biallelic alterations involving both a loss of the wild-type allele from a large interval genomic aberration and a mutant allele. The results for genes with biallelic hits were considered to be strong candidates for a loss-of-function involvement in cancer (Table 1).

### Identification of cancer drivers common to the primary tumor and metastasis

Both the primary and the metastasis contained cancer driver events that were likely to be critical for tumorigenesis in the context of the initial *CDH1* mutation (Table 1, Figure 2). In addition to the germline *CDH1* intronic mutation, the second *CDH1* allele had a somatic 77 bp genomic deletion of a portion of exon 9 that affects the downstream coding regions as well. The *CDH1* somatic mutation was identical in both the primary and metastatic gastric cancer genomes, demonstrating a common genetic origin and providing strong genetic evidence that this driver had a critical role in diffuse gastric tumorigenesis. Mutations affecting *CDH1* exon 9 that lead to loss of protein expression have frequently been detected in diffuse gastric cancer [18-20]. This exon’s amino acid sequence is a putative calcium-binding site that is likely important for receptor function.

The primary and metastatic tumor also shared biallelic splice donor site mutation (c.559 + 1G > A) of the fifth intron of *TP53* and a chromosome 17p LOH event encompassing the *TP53* locus (Additional file 1: Figure S1). The *TP53* splicing mutation interrupts RNA splicing [21] and is a previously reported cancer mutation [22,23]. The analyses of sporadic and inherited gastric cancers have identified *TP53* mutations that occur concurrently with *CDH1* mutation [24,25]. *CDH1* inactivation in gastric parietal cells does not induce gastric carcinoma, suggesting that loss of *CDH1* is insufficient for tumor initiation [26]. However, double conditional knockout of *CDH1* and *TP53* induces development of diffuse gastric carcinoma [26]. Interestingly, the genomic interval of the LOH event affecting the *TP53* locus was larger in the metastasis compared to the primary tumor. This could have occurred because of independent genomic instability events given the strong selection for biallelic loss of TP53 function.

### *FGFR2* is an actionable cancer driver exclusive to the primary gastric tumor

In the primary tumor, there was a six-fold genomic amplification of a region of chromosome 10 q arm and covered an interval of 1.66 Mb. Within this genomic regions was an oncogenic candidate driver *FGFR2* also referred to as the fibroblast growth factor receptor 2 (Figure 3c). This was confirmed with multiple methods including sequencing, array analysis, and validation by quantitative PCR. FGFR2 is a transmembrane receptor that acts as part of a key signal transduction pathway regulating tissue repair and embryonic development among a host of other functions [26].

**Figure 4:**
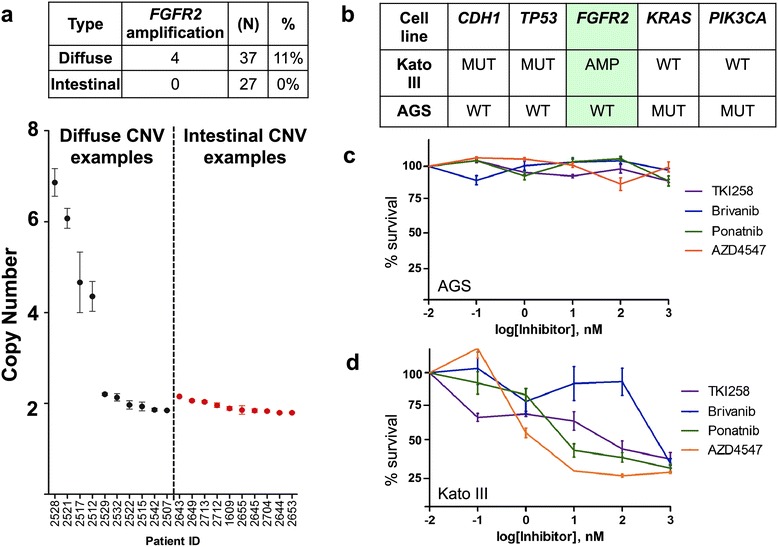
**Prevalence of** ***FGFR2*** **in human gastric tumors and its contribution to cellular proliferation. (a)** Sporadic gastric cancer samples were evaluated by quantitative digital PCR to determine FGFR2 genomic copy number. Black dots represent diffuse gastric cancers. Red dots indicate the intestinal subtype of gastric cancer. **(b)** Genetic characteristics of the AGS (*FGFR2* diploid) and KatoIII (FGFR2 amplified) gastric cancer cell lines are shown. **(c)** Percent survival for the AGS cancer cell line is shown with FGFR2 inhibitors of varying specificity. **(d)** The KatoIII diffuse gastric cancer cell line was treated with FGFR2 inhibitors of varying specificity. The Y-axis depicts percent survival *versus* the X-axis with log concentrations. In all panels, error bars represent standard error of the mean. The difference in percent cell survival between KatoIII and AGS cells was statistically significant (*P* <0.05) at the three highest concentrations of all drugs, except Brivanib which was only significant at the highest concentration.

To validate the prevalence of *FGFR2* amplification in diffuse versus intestinal gastric cancers, we analyzed 37 diffuse and 27 intestinal subtype primary gastric tumor samples with digital PCR [27]. Previously, we demonstrated that this method is profoundly sensitive for detecting copy number aberration even in the context of normal diploid DNA diluting tumor DNA. Our study demonstrated *FGFR2* amplification in four of 37 (11%) diffuse tumor samples, which was absent in the intestinal subtype samples (Figure 4a).

In support of its role as a candidate driver, *FGFR2* amplification is present in a number of gastric cancer cell lines [28,29] and subsequently reported in various gastrointestinal malignancies such as esophageal adenocarcinoma [30]. In addition, treatment of cancer cell lines with FGFR2-specific small molecule inhibitors or shRNAs leads to potent growth inhibition [28] suggesting a functional role for *FGFR2* amplification in the diffuse subtype.

### Functional analysis of the *FGFR2* driver in combination with *CDH1* and *TP53*

We identified two examples of a primary diffuse gastric cancer with co-occurrence of known and putative cancer drivers involving *CDH1*, *TP53*, and *FGFR2* as seen in the index patient. The first example included a diffuse gastric cancer sample that was among the gastric adenocarcinomas analyzed by TCGA. Using the cBio TCGA portal [10], we identified a patient (TCGA-BR-6803) who had a similar complement of genetic aberrations in *CDH1*, *TP53*, and *FGFR2*, all of which have been previously described in cancer as seen in the COSMIC cancer mutation repository. This included the following: a missense mutation in *CDH1* (D254Y) that has been described in three other cancers; a missense mutation (L130F) in *TP53* where mutations in this codon have been reported in 37 other cancers; the *FGFR2* amplification which we and others have identified in diffuse gastric cancer.

As the second example, we identified a human diffuse gastric cancer cell line, KatoIII, which has a similar composition of genetic aberrations affecting the same cancer genes as the primary tumor of our index patient. KatoIII has a *CDH1* mutation leading to an intronic sequence insertion in the mRNA [31,32], a *TP53* mutation leading to a complete gene deletion [33] and the *FGFR2* amplification [29] (Figure 4b). This cell line allowed us to assess the potential oncogenic role of the *FGFR2* amplification in the specific genetic context of *CDH1* and *TP53* mutations, similar to the index patient’s primary tumor.

To determine the contribution of FGFR signaling to neoplastic growth, we treated KatoIII cells with several FGFR2 small molecule tyrosine-kinase inhibitors (TKIs), including Brivanib, TKI258, Ponatinib, and AZD4547 [34]. As a control, we used the gastric cancer cell line AGS which is wild type for *FGFR2*, *CDH1*, and *TP53*, but has mutations in *KRAS* and *PIK3CA* [35] (Figure 4b). All FGFR2 inhibitors induced cell death in KatoIII but not AGS cells (Figure 4c and d). The most potent of these TKIs, AZD4547, has an IC_50_ of approximately 2 nM in KatoIII cells and 39,580 nM in AGS cells (Figure 4c and d). Each of the inhibitors demonstrated a statistically significant lower IC_50_ in FGFR2-amplified KatoIII cells compared to non-FGFR2-amplified AGS cells at all concentrations tested (Figure 4c and d).

In contrast, treatment of KatoIII and AGS cells with cytotoxic chemotherapeutic agents such as paclitaxel, 5-fluorouracil and carboplatin did not have a significant effect on either KatoIII or AGS lines, with similar IC_50_ identified in each (Additional file 1: Table S7). For AZD4547, the 20,000-fold difference in sensitivity to FGFR inhibitors suggests that FGF signaling is a critical driver to *CDH1*-initiated gastric cellular proliferation and this TKI represents a potential targeted therapy in diffuse subtype cancers harboring *FGFR2* amplifications.

### Biallelic inactivation of *TGFBR2* is exclusive to the ovarian metastasis

Genetic divergence was evident; the metastasis harbored its own unique subset of mutations and genomic aberrations. As we described, the metastasis had the same *CDH1* and *TP53* mutations as the primary tumor but lacked the *FGFR2* amplification found in the primary cancer site (Figure 3c). To eliminate the possibility that the absence of *FGFR2* amplification was related to a subpopulation not present in our original metastatic section, we performed a highly sensitive quantitative digital PCR on a separate geographic region from the metastasis (data not shown). This method has been previously been demonstrated to identify *FGFR2* copy number amplifications with high sensitivity and specificity, even in the context of diluted mixtures [27]. This independent analysis again confirmed that the *FGFR2* locus was not amplified in a separate region of the metastatic tumor.

The most striking event uniquely defining the metastasis was a *TGFBR2* deletion in exon 3 (Table 1). We looked for the presence of this somatic mutation among the normal and primary tumor sequence from the independent datasets (for example, whole genome, exome, and deep targeted resequencing). The MAF of the mutation among all of these sequencing datasets indicated exclusivity specific to the ovarian metastasis (Additional file 1: Table S5). The mutation was not found in any significant fraction among the primary and normal genomes.

*TGFBR2* encodes a receptor for the transforming growth factor β (TGF-β) pathway. While *TGFBR2* is mutated in numerous human cancers with particular prevalence in mismatch repair-deficient colon cancer [36], its functional relevance in gastric cancer is unknown. This particular deletion markedly reduces mRNA levels, presumably due to nonsense-mediated decay [37]. The metastasis also harbored a unique large genomic deletion of chromosome arm 3p encompassing the *TGFBR2* locus as shown by both CNV and LOH events, resulting in biallelic events affecting the wildtype *TGFBR2* alleles (Figure 3b).

*TGFBR2* exon 3 deletions are typically associated with colorectal tumors displaying microsatellite instability (MSI), a molecular marker for the loss of DNA mismatch repair (MMR). We assessed the primary and metastatic tumor for DNA mismatch repair defects. The primary tumor had normal immunohistochemical staining for the major DNA mismatch repair proteins MLH1, MSH2, PMS2, and MSH6. Neither the primary tumor nor metastasis exhibited elevated MSI at any of the diagnostic genetic markers (Additional file 1: Table S7). In addition, the patient had no germline, primary tumor or metastatic somatic mutations in the MMR genes.

We examined the cBIO TCGA dataset for gastric cancers classified by the Lauren histopathologic criteria as diffuse. Among the TCGA set, three of 79 diffuse gastric tumor samples had mutations in *TGFBR2*. This included two cancers in which there was biallelic loss of the wild-type allele [10]. These samples were MSI stable. The diffuse subtype samples with *TGFBR2* mutations include: a homozygous deletion (TCGA-BR-A4QM); biallelic mutations involving F442S and A426V in (TCGA-D7-6522); Q418 splice site mutation (TCGA-CD-8531). The examples of *TGFBR2* mutations existing in diffuse gastric cancers are supportive evidence for the potential role of *TGFBR2* as a driver.

### Other candidate cancer genes delineating the metastasis from the primary tumor

Additional candidate cancer genes were identified that distinguished the metastasis from the primary gastric tumor (Table 1). A novel predicted pathogenic mutation in *BMP7* was identified in the primary and metastatic tumor but the metastatic tumor had a unique copy neutral loss of heterozygosity event encompassing the entire chromosome arm 20 q including the *BMP7* locus. BMP7 (that is, bone morphogenic protein) interacts with the TGF- β pathway and has a well-studied role in osteoclast differentiation and bone development [38]. In addition, BMP7 expression has been correlated with tumor recurrence in gastric cancer [39].

Similarly, a novel *DOCK1* mutation was uniquely identified in the metastatic genome. DOCK1 regulates cell motility and migration and has been implicated in ovarian cancer tumorigenesis [40] (Additional file 1: Table S5). Another genomic amplification unique to the primary tumor occurred in the 5q22.3 locus (Additional file 1: Table S3). Among the 15 genes within the amplification locus, the major oncogenic-related cancer gene was *TRIM36* that is overexpressed in prostate cancer. It has been hypothesized its overexpression leads to chromosomal instability [41,42].

### *TGFBR2* knockdown in the context of *CDH1* and *TP53* is sufficient to induce metastatic diffuse gastric cancer in a primary gastric organoid murine model

Given the metastasis-specific, biallelic alteration of *TGFBR2*, we exploited our validated primary air-liquid interface murine gastric organoid system [12,13] to investigate if *TGFBR2* knockdown was sufficient to induce gastric cancer metastasis. Its consideration as a candidate was also suggested by the TCGA data. Previously, we observed that *Trp53* deletion and *Kras*^*G12D*^ induced pronounced *in vitro* dysplasia and invasion of gastric organoids with *in vivo* tumorigenicity upon subcutaneous implantation, but spontaneous metastasis was not seen by 50 days [13].

**Figure 5:**
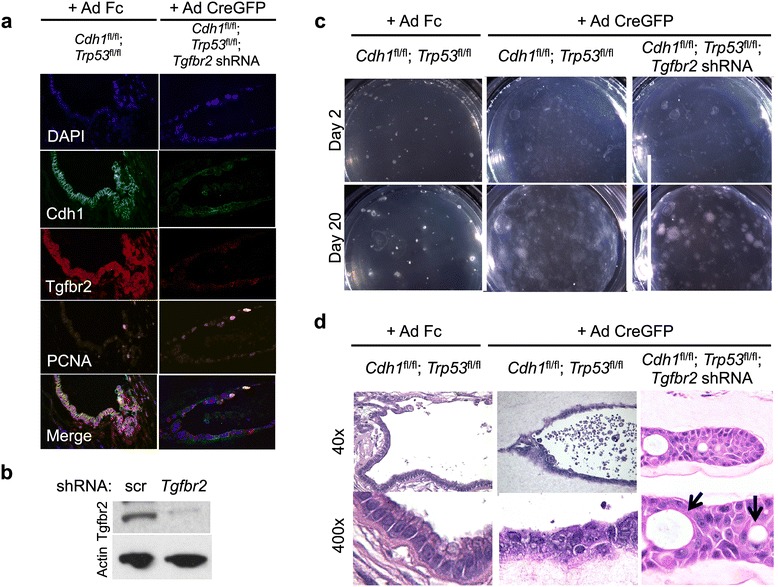
**Dysplastic epithelium in gastric organoids. (a)** Gastric organoid cultures were made from gastric tissue of neonatal mice harboring *Cdh1* and *Trp53* floxed alleles then subsequently infected with Fc-expressing adenovirus, or CreGFP-expressing adenovirus +/- retrovirus expressing shRNA against *Tgfbr2*. Images indicate immunofluorescence with nuclear DAPI staining and antibodies against CDH1, TGFBR2, or PCNA. Intrinsic GFP fluorescence from adenovirus CreGFP was abrogated by tissue passaging and subsequent formaldehyde fixation, and is not visible in immunofixation experiments. **(b)** GSM-06 murine gastric epithelial cells were infected with scrambled shRNA or an shRNA against murine *Tgfbr2* and lysates probed by western blotting with antibodies against TGFBR2 or β-actin. **(c)** Gastric organoid cultures were made from gastric tissue of neonatal mice harboring *Cdh1* and *Trp53* floxed alleles and subsequently infected with retrovirus expressing shRNA against *Tgfbr2*. Images are of cultured spheres at days 2 and 20. **(d)** Images represent H&E stained gastric organoids with the indicated genotypes taken at low (40×) or high power (400×).

Since both the primary and metastasis shared common *CDH1* and *TP53* mutations, primary gastric organoids were established from *Cdh1*^fl/fl^;*Trp53*^fl/fl^ neonatal mouse stomach. Gastric organoid infection with a control adenovirus (Ad Fc) encoding an immunoglobulin Fc fragment [43] resulted in gastric organoids with wild-type Cdh1 and Tp53, while adenovirus Cre-green fluorescent protein (Ad Cre-GFP) induced deletion of the floxed *Cdh1* and *Trp53* alleles, with Cdh1 and Trp53 loss confirmed by immunofluorescence (Figure 5a and Additional file 1: Figure S2), accurately modeling the *Cdh1* and *Trp53* loss common to both the primary and metastatic tumors. As we previously reported [12,13], Ad Fc-treated organoids with wild-type *Cdh1*/*Trp53* contained epithelial and mesenchymal components, accurately recapitulating *in vivo* stomach tissue architecture (Figure 5a and 5d).

To model the effect of the *TGFBR2* in metastatic oncogenesis, we infected the same *Cdh1*^-/-^;*Trp53*^-/-^ gastric organoids with retrovirus expressing shRNA against *Tgfbr2*, confirming *Tgfbr2* knockdown by immunofluorescence and Western blot analysis (Figure 5a and b). Likewise, gene expression of *Tgfbr2* was also reduced as determined by real time PCR (Additional file 1: Figure S3). The *Tgfbr2* shRNA did not grossly increase the growth rate of *Cdh1*^-/-^;*Trp53*^-/-^ gastric organoids over a 20-day period, possibly because of dominant effects of the *Cdh1* and *Trp53* deletions (Figure 5c). However, histologic analysis revealed that the resultant *Cdh1*^-/-^;*Trp53*^-/-^; *Tgfbr2* shRNA gastric organoids but not *Cdh1*^-/-^; *Trp53*^-/-^ controls demonstrated features of diffuse subtype gastric cancer. Severe dysplasia along with focal areas of invasion, signet ring formation, and nuclear pleomorphism were found throughout the analyzed organoids (Figure 5d).

**Figure 6:**
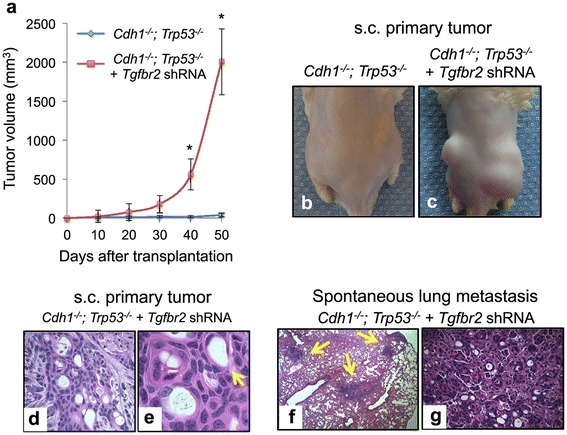
**Gastric organoid tumor explants.** Gastric organoids with the indicated genotypes are shown. **(a)** Tumor volumes were measured over time post-injection and plotted according to genotype of the driver combinations being tested. This includes *Cdh1* ^-/-^;*Trp53* ^-/-^ as shown in blue and *Cdh1* ^-/-^;*Trp53* ^-/-^;*Tgfbr2 shRNA* as shown in red. Error bars represent SEM. Asterisk (*) indicates *P* <0.01 for *Cdh1* ^-/-^;*Trp53* ^-/-^;*Tgfbr2* shRNA compared to *Cdh1* ^-/-^;*Trp53* ^-/-^ tumor volumes. **(b, c)** With different driver combinations, transformed gastric organoids were dissociated and subcutaneously (s.c.) injected into the flanks of immunodeficient NOG mice. Images indicated tumor growth at 30 days post injection. **(d, e)** Histological analysis of tumors confirms the presence of poorly differentiated adenocarcinoma with signet ring features, as indicated by the yellow arrows, only in the *Cdh1* ^-/-^;*Trp53* ^-/-^;*Tgfbr2 shRNA* organoids. After flank injections with dissociated organoids, histological analysis of murine lungs after 30 days revealed metastatic gastric adenocarcinoma with signet ring features at low **(f)** and high **(g)** magnification.

To examine potential *Tgfbr2* effects on *in vivo* metastasis, the *Cdh1*^*-/-*^*;Trp53*^*-/-*^*;Tgfbr2 shRNA* organoids *versus Cdh1*^*-/-*^*;Trp53*^*-/-*^ controls were disaggregated and injected subcutaneously into immunodeficient NOG mice. *Cdh1*^*-/-*^*;Trp53*^*-/-*^ organoids produced extremely slow but detectable tumor growth by day 50 as we previously documented [13] (Figure 6a and b). In contrast, *Cdh1*^*-/-*^*;Trp53*^*-/-*^*;Tgfbr2 shRNA* gastric organoids exhibited robust *in vivo* tumorigenicity (Figure 6a and c). Notably, *Cdh1*^*-/-*^*;Trp53*^*-/-*^*;Tgfbr2 shRNA* primary tumors exhibited a poorly differentiated adenocarcinoma histology with signet ring features as occurs in diffuse gastric cancer (Figure 6e to g). Immunofluorescence analysis confirmed loss of Cdh1 and Tgfbr2 knockdown (Figure 5a).

Evaluation for distant disease confirmed the presence of pulmonary metastases in NOG mice harboring *Cdh1*^*-/-*^*;Trp53*^*-/-*^*;Tgfbr2 shRNA* tumors, comprised of poorly differentiated adenocarcinoma with signet ring features (Figure 6f, g). Metastatic tumors were located in the lungs bilaterally, were grossly observable upon dissection and had similar histologic appearance to diffuse gastric cancer. Overall, these studies support the role of *Tgfbr2* as a putative tumor suppressor gene in diffuse gastric cancer, demonstrate successful *in vitro* conversion of primary gastric tissue to metastatic adenocarcinoma, and reveal the utility of a primary gastric organoid system for functional validation of candidate metastasis drivers.

## Discussion

To address the question of identifying the genetic drivers of diffuse gastric cancer metastasis, we performed an extensive genome sequencing analysis of the metastatic evolutionary process. This involved sequencing of a matched gastric primary and subsequent metastasis from the same patient. We leveraged the unique Mendelian genetics of a HDCG proband as an ‘experiment of nature’ to delineate essential cancer drivers in diffuse gastric cancer.

Our genomic analysis revealed *FGFR2* amplification exclusive to the primary gastric tumor and not present in the metastasis. Our results fully confirm several descriptions of FGFR2 amplification, as well as increased sensitivity of FGFR2-amplification positive cell lines such as KatoIII to small molecule FGFR inhibitors [44-47], with accompanying implications for FGFR2-targeted treatment [48]. The striking absence of *FGFR2* amplification in the metastasis in the context of the common somatic *CDH1* and *TP53* mutations argues strongly for a tumor evolutionary divergence.

The functional validation of the metastatic potential of *Tgfbr2* knockdown in our well-validated air-liquid interface gastric organoid method [12,13] provides the first demonstration that *TGFBR2* functions as a bona fide metastasis suppressor gene in diffuse gastric cancer. Homozygous *TGFBR2* deletion is also present in a subset of TCGA gastric cancers [10] which are largely comprised of non-metastatic tumors. It will be interesting to evaluate whether *TGFBR2* alterations are more prevalent in gastric metastases, such as to ovary or other sites. Furthermore, the functional relevance of other potential loci undergoing alteration in these samples merits additional exploration.

Our study also describes the first successful *in vitro* conversion of primary gastric tissue to metastatic gastric adenocarcinoma, suggesting the general applicability of the organoid method to the functional validation of gastric cancer loci involved in progression and/or metastasis. As shown here, such three-dimensional organoid-based functional validation strategies can potentially combine both the experimental tractability of two-dimensional culture of transformed cell lines with the accurate tissue ultrastructure and stromal components of transgenic mouse systems.

## Conclusions

Exclusive *FGFR2* and *TGFBR2* genetic aberrations delineated the evolution of metastatic recurrence. Our finding may have implications for targeted cancer therapy. For example, the index patient in this study may have conceivably responded to a FGFR2 inhibitor based on *FGFR2* amplification in the primary tumor. However, the patient’s metastatic recurrence did not harbor this same *FGFR2* amplification, suggesting that treatment with a therapeutic small molecule inhibitor may not have had a discernible biological effect on the patient’s metastatic disease. In the precision management of individuals with metastatic cancer, one may need to account for the genetic heterogeneity and subsequent variation in cancer biology that differentiates metastatic sites from the primary tumor before the initiation of targeted therapy.

Although a comprehensive knowledge of the metastatic process is crucial for improved cancer treatment, the driver events underlying metastatic spread are unfortunately poorly understood [49]. Our paucity of knowledge regarding metastasis is further compounded by the relative omission of metastatic samples in large-scale genomic cancer surveys such as TCGA. This study is an initial effort to further address these questions about the genetics and biology of metastatic evolution by integrating genomic sequencing analysis with *in vitro* validation of clonal-specific candidate drivers.

## Materials and methods

### Cancer samples

This study was conducted in compliance with the Helsinki Declaration. The institutional review board (IRB) at Stanford University School of Medicine approved the study protocols (11886 and 19071). For all patients cited in this study, we obtained informed consent to conduct research and publish the results. Samples were obtained from the Stanford Cancer Institute Tissue Bank. Frozen tissue sections were prepared from each tumor and hematoxylin-eosin (H&E) staining was performed on a single section. We estimated overall tumor composition that generally was approximately 50% or greater for most samples. Tumors were macro-dissected to increase tumor cellularity and processed for genomic DNA. Full details are in the Additional file 1: Methods.

### Sample preparation for whole genome, exome, and targeted resequencing analysis

Genomic DNA was extracted from blood, normal gastric tissue and tumor samples using the E.Z.N.A. SQ DNA/RNA Protein Kit (Omega Bio-Tek, Norcross, GA, USA). Concentrations of genomic DNA were determined with a Nanodrop instrument (Thermo Scientific, Wilmington, DE, USA). Genomic DNA from matched normal and cancer tissue were then used for creating sequencing libraries. DNA from peripheral leukoctyes was used for the Affymetrix SNP array.

From each sample, we fragmented 4 μg of genomic DNA with a Covaris instrument (Covaris, Woburn, MA, USA). Illumina TruSeq Paired End libraries were constructed from double stranded, fragmented DNA per Illumina’s standard protocol (Illumina, San Diego, CA, USA). The amplified material was recovered with a Qiaquick (Qiagen) column according to the manufacturer’s instructions, except the DNA were eluted in 50 μL water. The sequencing library DNA was quantified using the NanoDrop-1000 and the library was evaluated with an Agilent Bioanalyzer 2100 (Agilent, Santa Clara, CA, USA) using a DNA1000 chip. The mean library fragment size was found to be 300 bp and these libraries were used for whole genome sequencing. For exome capture hybridization, we used Nimblegen SeqCap version 2 enrichment assay (Roche-Nimblegen, Madison, WI, USA). The methods were according to the NimbleGen’s SeqCap EZ Exome Library SR User’s Guide v2.2. Following the final amplification reaction, we purified the exome libraries using a Qiaquick column (Qiagen, Valencia, CA, USA) per the manufacturer’s recommended protocol.

### Cancer genome sequencing

See Additional file 1 for complete details regarding the whole genome, exome, and targeted resequencing data analysis. This includes information about the targeted resequencing process, variant calling, allelic frequency determination, and mutation interpretation The oligonucleotide sequences for deep targeted resequencing are listed in Additional file 2.

### *FGFR2* amplification analysis from diffuse and intestinal gastric cancers

Quantitative PCR was performed using the Bio-Rad QX100 droplet digital PCR (ddPCR) system (Bio-Rad, Pleasanton, CA, USA). We used a standard set of *FGFR2*-specific TaqMan primers and probes (Life Technology, Foster City, CA, USA) compared with standard references using an ultra-conserved region on chromosome 1. Briefly, TaqMan PCR reaction mixtures were assembled using 2× ddPCR Supermix for probes, 20× assays (18 μM primers and 5 μM probe) and restriction digested DNA samples (Biorad). To assess *FGFR2* copy number, 125 ng of each tumor DNA sample was digested with 1.25 units of BsaJI (NEB) in 15 μL for 1 h at 60°C. The digests were diluted 1.67-fold to 25 μL with nuclease free water then 25 ng (5 μL) was assayed per 20 μL ddPCR reaction. *FGFR2* assay sequences were (forward primer) 5’-GGCTGGCTGCTGAAGTCT-3’, (reverse primer) 5’-CTTAATCGCCTGTATGGTGGTAACA-3’, and (probe) 5’-FAM-TCTTGGTCGTGTTCTTCATTCGGCACAG-BHQ1-3’. The *FGFR2* assay was duplexed with a standard reference sequence on Chromosome 1. This standard reference assay used the following primers: (forward primer) 5’-TGAGGGATTCGGCAGATGTTG-3’, (reverse primer) 5’-CTGAAAGGCTGGACTTGACAGA-3’, and (probe) 5’-VIC-ACTGTGTGCTGGACCT-MGB-3’. All assay primers were ordered from Integrated DNA Technologies. Thermal cycling conditions were 95°C 10 min (1 cycle), 94°C 30 s and 60°C 60 s (40 cycles), 98°C 10 min (1 cycle), and a 12°C hold. *FGFR2* copy number per cell was estimated as the ratio of the *FGFR2* and *RPP30* concentrations multiplied by two to account for the two copies of *RPP30* that are expected per diploid genome. Analysis of the ddPCR data was performed using the CNV mode of the QX100 analysis software (version 1.2.9.0). Quadruplicate ddPCR wells were analyzed for each sample.

### FGFR2 inhibitor sensitivity assay

KatoIII cells (HTB-103, ATCC) and AGS cells (CRL-1739, ATCC) were grown in Dulbecco’s Modified Eagle Medium (DMEM), supplemented with 10% fetal bovine serum and 100 U/mL of Pen Strep Glutamine (Gibco). All cells were cultured at 37°C in a humidified atmosphere and 5% CO_2_. Survival of KatoIII and AGS cells was determined using the WST-1 Proliferation Assay (Roche). We tested multiple FGFR inhibitors including TKI-258, Brivanib (BMS-540215), Ponatnib (AP24534), and AZD4547 (Selleck Chemical). Cells were seeded at a density of 2 × 10^4^ cells/well in 96-well microtiter plates, 100 μL medium/well and maintained 18 h for attachment. Afterwards, we treated the cultures with varying concentrations of each drug diluted in DMSO. After 30 h incubation, 10 μL of WST-1 reagent was added followed by 1 h at 37°C. The cleavage of tetrazolium salt (WST-1) into a visible formazan by viable cells was spectrophotometrically measured using a reference wavelength of 450 nm. Each test was performed in triplicate. Percentages of cell survival were calculated as follows:% cell survival = (absorbance of treated cells/ absorbance of cells with vehicle solvent) × 100. The half inhibitory concentration (IC_50_) was calculated with a non-linear regression from the dose–response curve.

### Mismatch repair protein immunohistochemistry

Mismatch repair protein immunohistochemistry was performed on the primary diffuse gastric tumor using the standard streptavidin-biotin-peroxidase procedure. Primary monoclonal antibodies against MLH1 (clone G168-728, 1:200, BD PharMingen, San Diego, CA, USA 1:200), MSH2 (clone FE11, 1:100, Oncogene Research Products, Cambridge, MA, USA), MSH6 (clone 44, 1:200, BD Transduction, San Jose, CA, USA) and PMS2 (clone MRQ-28, 1:10, Cell Marque, Rocklin, CA, USA) were applied to formalin-fixed, paraffin embedded sections four microns thick. The sections were deparaffinized in xylene, and rehydrated through graded alcohols to distilled water before undergoing antigen retrieval by heat treatment in either citrate solution pH 6.0 (MLH1, PMS2, and MSH2) or EDTA solution pH 9.0 (MSH6). An automated detection using a Leica Bond Autostainer (Leica, Buffalo Groove, IL, USA) was employed. Normal expression was defined as nuclear staining within tumor cells, using infiltrating lymphocytes as positive internal control. Negative protein expression was defined as complete absence of nuclear staining within tumor cells in the face of concurrent positive labeling in internal non-neoplastic tissues.

### Gastric organoid cancer modeling in mice and functional analysis

All procedure involving animal were approved the Stanford University Administrative Panel on Laboratory Animal Care and was fully compliant with the USDA Animal Welfare Act, and our Assurance of Compliance with the PHS Policy on Human Care and Use of Laboratory Animals. Air-liquid interface organoid culture was performed as described [11,12].

*Cdh1*^flox/flox^;*Trp53*^flox/flox^ mice were generated by crossing *Cdh1*^flox/flox^ mice, obtained from Jackson Laboratory, and *Trp53*^flox/flox^ mice, kindly provided by Dr. Anton Berns [50] NOD.Cg-Prkdc^scid^ Il2rg^tm1Sug/JicTac^ mice were obtained from Taconic Farms, Inc. We dissected stomachs from neonatal mice (age P1-10) and washed them in cold PBS to remove all luminal contents. We extensively minced either large 25% sections or any entire neonatal stomach per dish and embedded the minced tissues in a 3D collagen gel using a double-dish air-liquid interface culture system as previously described [11]. To maintain the organoids, we applied fresh medium (F12, 20%FCS, gentamicin 50 ug/mL) every week.

*Tgfbr2* shRNAs were obtained from Origene (catalog TG516186). Retroviral plasmids were cotransfected with pCL-Eco into 293 T cells by Lipofectamine2000 (Invitrogen). Retroviral supernatants were collected 48 and 72 h post-transfection and concentrated by PEG-it virus precipitation solution (5×, System Biosciences). Virus titer was determined by infection of NIH3T3 cells and FACS analysis of GFP positive cells 48 h post infection. *Cdh1*^flox/flox^;*Trp53*^flox/flox^ gastric organoids were infected at day 0 with adenovirus Ad Cre-GFP (University of Iowa Vector Core) or control adenovirus Ad Fc [43] encoding a mouse immunoglobulin IgG2α Fc fragment by layering viral particles (10^9^ pfu) suspended in 500 μL culture media over the top of the collagen matrix containing primary tissue. For retrovirus infection of secondary organoids, primary organoids at 14 to 20 days of growth were recovered from collagen gel by collagenase IV (Worthington) incubation followed by 0.05% trypsin/EDTA incubation to dissociate organoids into a single cell suspension. Following extensive washing with 10% FBS to inactivate collagenase/trypsin, cells were pelleted by centrifugation and incubated with retroviral particles (2 μL of 10^8^ pfu/mL) encoding Tgfbr2-shRNA in the presence of growth medium and TransDux (System Biosciences) at room temperature for 60 min before serial replating into 3D collagen gel air-liquid interface culture.

Samples were fixed with 4% paraformaldehyde overnight, paraffin-embedded, sectioned, and sections stained by H&E for initial histology analysis. Further immunohistochemistry analysis, used the following antibodies: PCNA (1:300; Invitrogen), CDH1 (1:300; BD Biosciences Pharmagen), TGFBR2 (1:250; Abbiotec), p53 (1:100; Santa Cruz). Cell lysates of mouse gastric culture cell or GSM-06 cells transfected with *Tgfbr2*-shRNA-GFP were immunoblotted with TGFBR2 (1:2,000, Abbiotec) and β-actin (1:2,000, Abcam).

Cells from gastric organoids were collected from the air-liquid interface collagen gel by disaggregation with collagenase IV (Worthington). For transplantation, 400,000 cells per mouse flank were mixed with matrigel (50% Matrigel, 10%FCS, 40% F12, 100 μL of Matrigel mixture per = mouse) and injected into NOD.Cg-Prkdc^scid^ Ilr2rg^tm1Sug/JicTac^ mice. Mice were sacrificed after day 50, after which tumors were dissected and examined by H&E staining. *P* values were determined using a two-tailed Student’s t-test assuming unequal variances. A *P* value of 0.05 was considered significant.

### Data availability

The data from this study have been submitted to the NCBI Sequence Read Archive under the accession number SRP044347.

## Additional files

Additional file 1:
**Supplementary Methods and Data.** **Table S1.** Summary of normal, tumor, and metastasis exome and whole genome sequencing coverage. **Table S2.** Single nucleotide variant and small indel calling from genome sequencing datasets. **Table S3.** Summary of the validated cancer copy number variations and loss-of-heterozygosity (LOH) events. **Table S4.** Summary of the cancer rearrangement events. **Table S5.** Validated cancer mutations and analysis of mutant allelic frequency. **Table S6.** Microsatelllite genetic markers assessed for microsatellite instability. **Table S7.** Gastric cancer cell line IC50s for different gastric cancer cell lines. The IC50s of cytotoxic chemotherapies (paclitaxel, carboplatin, 5-fluorouracil) or an FGFR2 small molecule tyrosine kinase inhibitor (AZD4547) are listed. **Figure S1.** Biallelic events in the *TP53* loci and gene. **Figure S2.** Deletion of *Cdh1* and *Trp53* expression in gastric organoids. **Figure S3.** shRNA-mediated knockdown of *Tgfbr2* demonstrate by real time PCR [16,17,23,51-74]. Additional file 2: Table S8. Mutation targeted sequencing study.

## Abbreviations

Ad: Adenovirus
BMP: Bone morphogenic protein
CNV: Copy number variation
ddPCR: Droplet digital PCR
EMT: Epithelial mesenchymal transition
*FGFR2*: Fibroblast growth factor receptor 2
GFP: Green fluorescent protein
H&E: Hematoxylin-eosin
HDGC: Hereditary diffuse gastric cancer
indel: Insertion-deletions
IRB: Institutional review board
LOH: Loss of heterozygosity
MAF: Minor allele frequency
Mb: Megabase
SNP: Single nucleotide polymorphism
TCGA: The Cancer Genome Atlas
*TGFBR2*: Transforming growth factor-β receptor 2
TKI: tyrosine kinase inhibitor
