# Supplementary material for: Metastatic tumor evolution and organoid modeling implicate TGFBR2 as a cancer driver in diffuse gastric cancer
Source: Genome Biol. 2014 Aug 27;15(8):428. doi: 10.1186/s13059-014-0428-9 (PMC4145231; doi:10.1186/s13059-014-0428-9)
Supplement: Additional file 1: — Supplementary Methods and Data. Table S1. Summary of normal, tumor, and metastasis exome and whole genome sequencing coverage. Table S2. Single nucleotide variant and small indel calling from genome sequencing datasets. Table S3. Summary of the validated cancer copy number variations and loss-of-heterozygosity (LOH) events. Table S4. Summary of the cancer rearrangement events. Table S5. Validated cancer mutations and analysis of mutant allelic frequency. Table S6. Microsatelllite genetic markers assessed for microsatellite instability. Table S7. Gastric cancer cell line IC50s for different gastric cancer cell lines. The IC50s of cytotoxic chemotherapies (paclitaxel, carboplatin, 5-fluorouracil) or an FGFR2 small molecule tyrosine kinase inhibitor (AZD4547) are listed. Figure S1. Biallelic events in the TP53 loci and gene. Figure S2. Deletion of Cdh1 and Trp53 expression in gastric organoids. Figure S3. shRNA-mediated knockdown of Tgfbr2 demonstrate by real time PCR [16,17,23,51-74]. [file 13059_2014_428_MOESM1_ESM.pdf]

**Supplementary Methods and Data**

# Metastatic tumor evolution and organoid modeling implicate *TGFBR2* as a cancer driver in diffuse gastric cancer

**Authors:**

Lincoln D. Nadauld[1,2], Sarah Garcia[2], Georges Natsoulis[1], John M. Bell[2], Laura Miotke[1], Erik S. Hopmans[2], Hua Xu[2], Reetesh K. Pai[3], Curt Palm[2], John F. Regan[4], Hao Chen[5], Patrick Flaherty[2], Akifumi Ootani[6], Nancy R. Zhang[7], James M. Ford[1], Calvin J. Kuo[6,§], Hanlee P. Ji[1,2,§]

**Institutions:**

[1]Division of Oncology, Department of Medicine, Stanford University School of Medicine, Stanford, CA, 94305, United States

[2]Stanford Genome Technology Center, Stanford University, Palo Alto, CA, 94304, United States

[3]Department of Pathology, University of Pittsburgh Medical Center, Pittsburgh, PA, 15213, United States

[4]Bio-Rad, Inc., Pleasanton, CA, 94566, United States

[5]Department of Statistics, Stanford University, Stanford, CA, 94305, United States

[6]Division of Hematology, Department of Medicine, Stanford University School of Medicine, Stanford, CA, 94305, United States

[7]Department of Statistics, The Wharton School, University of Pennsylvania, Philadelphia, PA, 19104, United States

**[§] Corresponding authors:**

Hanlee P. Ji
Division of Oncology, Department of Medicine – Stanford University School of Medicine
CCSR 1115, 269 Campus Drive
Stanford, CA 94305-5151
Email: genomics_ji@stanford.edu
Phone: 650-721-1503
Fax: 650-725-1420

Calvin J. Kuo
Division of Hematology, Department of Medicine
Stanford University
CCSR 1155, 269 Campus Drive
Stanford CA 94305-5151
Email: cjkuo@stanford.edu

**INDEX**


**Supplementary Methods** (pp 1 – 11)

**Supplementary Tables*** (pp 12 – 21)

**Supplementary Figures and Legends** (pp 22 - 24)


* **Table S8** is available as a separate file.

## SUPPLEMENTARY METHODS

### Index patient

The patient (III-1) had a family history of gastric cancer consistent with hereditary diffuse gastric cancer (HDGC) syndrome [51]. The family's pedigree is shown in **Figure 1**. The patient's sister (III-2) had diffuse gastric cancer at the age of 46 years and was also found to be a carrier of the familial *CDH1* mutation. The maternal uncle had pancreatic cancer (II-2). The patient's maternal aunt (II-3) was diagnosed with gastric carcinoid at the age of 56 years and had a recurrence at 62 years.

### Whole genome and exome sequencing, alignment and coverage analysis

Exome libraries were prepared with the Roche EZ SeqCap exome enrichment probes v2.0 [52]. We used an Illumina HiSeq 2000 instrument with 100 base reads. Whole genome and exome sequencing libraries were clonally amplified through cluster-generation on an Illumina cBot using paired end flowcells and Illumina TruSeq v2 chemistry. Using PicoGreen assay (Invitrogen, San Diego CA) for quantitating the amount of library, we prepared samples for input according to the Illumina cBot User Guide. We loaded sample sequencing libraries at a final concentration of 1.5 pM; we generated an average cluster density of 450K clusters/mm$^2$. The clustered flowcell was sequenced on an Illumina HiSeq 2000 for 2 X 100 cycle reads with indexing, using Illumina TruSeq v2 reagents.

Sequence reads aligned to the human genome version hg19 using Burrows-Wheeler Aligner (BWA) [53]. The Genome Analysis Toolkit (GATK) was used to determine overall sequencing coverage for both whole and exome sequencing data sets [54]. Sequencing coverage is listed in **Table S1**. For the exome capture, the dataset used to determine the overlaps was the annotation BED file for the NimbleGen SeqCap EZ platform (v2)

(www.nimblegen.com/products/seqcap/ez/index.html) for the exome capture. The library covers over 30,000 genes across a 36 Mb region, and the probes cover a total of 44.1 Mb.

**Variant calling from cancer exomes and whole genome sequencing**

Somatic single-nucleotide variants (SNVs) and insertion deletions (indels) were called using the variant caller programs GATK (vs 1.0.577) [54] and Varscan [53]. Both variant callers were run to improve our sensitivity of detection. Varscan was run with the suggested parameters for tumor-normal pair comparison [55]. For Varscan calls, we required evidence on both forward and reverse strands and applied a p-value cut-off of 0.001.

We used the following parameters for analysis with GATK [56]. Base recalibration relied on CountCovariates and TableRecalibration functions. Four different covariates settings were used for the "CountCovariates" option of CountCovariates, ReadGroupCovariate, QualityScoreCovariate, CycleCovariate and DinucCovariate. The output of CountCovariates was used with GATKs TableRecalibration to recalibrate quality scores. The default options were used for this application. In between steps, BAM sequence files were resorted and indexed with Picard sequence analysis suite (http://picard.sourceforge.net/index.shtml) as needed. GATK's UnifiedGenotyper was used for variant calling with parameters recommended by the Broad Institute's best practices for variant discovery guidelines for coverage > 10 (-stand_call_conf 30.0 -stand_emit_conf 10.0). SNVs and indels were called together using the "BOTH" option for the "glm" parameter of the UnifiedGenotyper. Filters were applied to flag poor quality/alignment artifact SNVs. Filters that were applied were for SNV clusters (--clusterWindowSize 10) and hard to validate (MQ0 >= 4 && ((MQ0 / (1.0 * DP)) > 0.1), strand bias (SB >= -1.0) and high depth (DP > 50 for genome, DP > 80 for exome). From the VCF output files we required a Phred scaled probability score of greater than 30 and a GATK filter

setting of “PASS”. We used the BED file for exome coverage within 100 bases of an exon boundary to limit variants to coding and adjacent regions.

For the next step post-variant calling, we filtered out the germline variants from cancer-specific mutations identified among the primary and metastatic cancer genome. Cancer-specific SNV and indel mutations were additionally categorized by potential functional relevance. For the primary gastric tumor, we aggregated the mutation results between the exome and whole genome sequencing. Not surprisingly, we found that the exome sequencing produced higher quality calls from exon regions. Whole genome sequence was useful for delineating mutations outside of exons. Given the lower coverage of the whole genome sequencing of the metastatic tumor, we exclusively relied on the exome sequencing for cancer mutation analysis. Our results are listed in **Table S2.**

All filtered GATK and Varscan calls were annotated using SeattleSeq131 (http://snp.gs.washington.edu/SeattleSeqAnnotation134/) to identify single nucleotide polymorphisms (SNPs) versus novel variants. Part of this annotation includes the comparison of variant positions with dbSNP130 [57, 58] and variants generated from the 1,000 Genomes Project [59]. We considered tumor-specific, novel variants overlapping with coding sequences, splice sites and RNA genes as potential mutations and these candidates were validated as described later.

Of the total variants detected in whole genome analysis by GATK, 92% of SNVs and 5% of indels were described in dbSNP130. Similarly, 94% of SNVs called by GATK in the exome data, and 6% of indels were present in dbSNP130. In the exome data, GATK predicted 49 non-germline, somatic variants present in both tumor and metastasis and 49 metastasis-specific variants. Varscan predicted 94 non-germline, somatic variants present in both tumor and

metastasis and 160 metastasis-specific variants. Given that the concordance between SNP array genotypes and GATK filtered calls was 99% in the normal sample, we generally used GATK calls and included a subset of Varscan mutation calls not identified from GATK for subsequent validation with an independent targeted resequencing analysis.

**Analysis of chromosomal allelic imbalance and copy number variation**

We used several methods to assess loss-of-heterozygosity (LOH) and somatic copy number variation (CNV) from the whole genome sequencing data of the cancer samples. For assessment of LOH events **(Figure 2; Additional file 1, Figure S1)**, we used a modification of the method employed originally by Solomon et al. [60] and more recently by Bettegowda et al. [61]. Our analysis relied on minor allelic frequency (MAF) data. The MAF is a ratio comparison of allelic read depths from heterozygous SNVs identified from the normal genome compared to the same position from the tumor. First, from each genome the GATK SNV calls are filtered to remove low quality data (quality < 30). Using read depths available from the VCF outputs for each of these heterozygous SNPs, we determined the MAF value by dividing the minor allele frequency over the major allele frequency for each genome (e.g. normal or cancer). For graphic display, we used a smoothed MAF value based on a window average of 10 contiguous SNPs from each genome. Cancer-related allelic imbalance alterations were based on deriving the log2 value of the cancer MAF over the normal MAF.

For statistical calling of the genomic intervals of specific LOH events, we first employed the analysis method published by Chen et al. on SNVs determined from the whole genome sequencing analysis of the two cancer genomes [62]. The associated R package for this analysis method is available under the project name "PSCN" at the following URL (http://r-forge.r-project.org/). The input data was the same as used in visualization and graphics. This data set involved 2.1 million SNPs that were noted to be heterozygote in the normal genome.

Our analysis involved a comparison with the same positions in the primary or metastatic tumor genomes. The PSCN algorithm was applied one chromosome at a time or applying the method to the whole genome without separating by chromosomes. We used the GATK-based read counts for the each allele of the SNP comparing the tumor to the normal sample to determine the intervals of allelic imbalance. Chen et al.'s method is also able to discriminate allelic imbalances even in the context of intermixed normal genomic DNA.

To determine somatic copy number alterations and the affected genomic intervals from whole genome sequencing data, we also used the published SeqCBS method [63]. This analysis method was informative in distinguishing LOH events attributable to genomic deletions versus copy number neutral alterations. SeqCBS uses circular binary segmentation (CBS) to find regions whose coverage differs significantly from neighboring regions [63]. The software implementation is available as an open-source R package named SeqCBS (http://cran.r-project.org). The CNV analysis used an R script that reads a configuration file listing the sequence data sets to be compared, namely the case (normal) versus the control (cancer). We also conducted an analysis comparing the primary versus the metastatic tumor as additional evidence of CNV differences. The algorithm then performs the segmentation on these two files, compares them, and produces both local and whole-chromosome CNV plots **(Figure 2, Additional file 1; Figure S1)**. For any such region, there is a general test statistic and a relative gain or loss copy number value. Generally, we required a test statistic > 1,000 as a basic cutoff and a copy number value of greater than 2.5 or less than 1.00 as our thresholds for marking an event as a significant amplification or loss. We determined the genes within CNV intervals specific to the primary or metastatic tumor.

### Cancer genome inter-chromosomal translocations, intra-chromosomal rearrangements and other structural variations

On the primary, metastatic and normal genome sequence, we used the Breakdancer program [64] to assess for inter-chromosomal translocations, large indels, rearrangements and other structural variants (SVs). For inter-chromosomal translocations, we performed separate primary tumor/normal tissue and metastatic tumor/normal tissue comparisons using breakdancer_max. To be considered as a potential variant, we required an anchor sequence of 20 base pairs on each side of a rearrangement breakpoint (breakdancer_max -t -s 20 -r 10 configfile). We also filtered out implausible cases (e.g. involving Y in female), required a minimum number of reads (20 for an individual genome finding or 10 common to two different genomes) and eliminated calls seen in the normal germline genome. For cancer-specific, intra-chromosomal events such as large genomic deletions, we required at least 20 reads to cover the putative breakpoint of the event that were seen in the primary or metastatic cancer but not the normal germline genome. As a final filter, we eliminated putative SVs where the anchor sequence occurred in highly repetitive sequences that were a potential source of mapping errors.

We also employed an integrated structural variation (SV) analysis pipeline [68] to identify rearrangements and other structural variants. This analysis pipeline relies on several programs to identify SVs: Pindel [66], CNVnator [67], BreakSeq [68] and BreakDancer [64]. HugeSeq calls a rearrangement if it is identified by at least two algorithms with at least 50% overlap of the genomic coordinates for the variants called. We separated the SV calls into high-confidence and low-confidence calls. As a default, this analysis generates cancer-specific calls by taking the set of variants called in the cancer by at least two algorithms and subtracting out those variants called in the normal tissue by at least two algorithms. We defined as high-confidence the tumor-specific SVs called by two algorithms in the primary tumor that were not identified in the normal genome. Filtering putative translocations whose anchors occur in repetitive regions left only three candidate translocations. All of these were found to occur either in normal only or in both normal and tumor and hence are not somatic. This included a number of candidates

including 146 deletions, 93 insertions, and 11 inversions. In total, 66 SVs were chosen for separate validation based on their proximity to coding regions. This validation set included all 43 putative events overlapping exons (17 high-confidence) and an additional 23 events overlapping non-exonic elements of genes involved in cancer (4 high confidence).

**Targeted resequencing and validation of cancer mutations and rearrangements affecting candidate cancer genes**

From patient 525, we identified a total of 345 SNVs, indels, and rearrangements overlapping with coding and regulatory regions and were in the primary and/or metastatic tumor. These cancer mutations and genomic aberrations were chose for independent targeted resequencing validation. As a general quality control, we included twelve control SNVs at random from a list of heterozygous germline SNVs variants present in both the tumor and the normal tissue. All but one of the randomly chosen control SNVs were in dbSNP.

The targeted resequencing assay as fully described by Myllykangas et al. [69]. Target-specific probe sequences were chosen flanking the target variant within approximately 150 to 300 bases from the target variant. In the case of SVs we chose probe sequences flanking the putative breakpoint. Our capture probe sequences were placed on both the forward and reverse strands flanking the target and filtered to eliminate sequence following in repeats. The specific oligonucleotide sequences are listed in **Table S8**. The free oligonucleotides for primer probes were synthesized and pooled. Targeted resequencing on these mutations was conducted as previously described [69]. We used 1 μg of genomic DNA from patient 525's primary tumor, metastatic tumor and normal genomic DNA from peripheral blood. Genomic DNA was randomly fragmented and processed to become OS-Seq libraries for targeted resequencing. After completion of the capture step, we carried out the routine Illumina flow cell preparation and sequencing. For the three samples (normal, primary tumor and metastasis) we generated 60 by

60 paired-end cycles on an Illumina Genome Analyzer IIx using vs. 4 sequencing reagents (Illumina). Image analysis and base calling were performed using the SCS 2.8 and RTA 2.8 software (Illumina).

Sequence reads were aligned to the human genome version human genome build hg19 using BWA [53]. For the de-multiplexing of indexed lanes, we generated an index of the 7-base tags using the base-call file to assign reads to the correct sample. The targeted sequencing reads generated from all 345 targeted variants were subjected to visual inspection with the Integrated Genome Viewer (IGV) [70] and variant calling with Varscan. The vast majority of the variants not passing visual inspection were excluded due to the presence of multiple reads supporting the variant in the normal blood, representing false negative germline calls

For structural variants, we used reads from the putative breakpoints that had a Phred-like score greater than 25. We aggregated the sequence reads for each rearrangement loci, eliminated reads that matched the reference genome and then conducted a localized assembly using Velvet [71] with the remaining reads. Parameters for Velvet included a hash length of 19, a contig length-minimum of 50 and a contig coverage depth minimum of 4. The generated Velvet contigs were aligned with megablast and filtered based on location on the correct chromosome, discontinuous sequence alignment starting from the breakpoint and appearance only in the cancer genome. We validated rearrangements based on contigs demonstrating clear breakpoint after this analysis.

**Cancer gene mutation and genomic aberration interpretation**

After targeted resequencing validation of specific mutations and genomic aberrations (e.g. CNVs), the confirmed cancer-specific variants were assessed their potential impact on protein function, mutations occurring in coding sequences were evaluated by SIFT {Kumar, 2009 #308}

and PolyPhen2 version 2.1.0 [16]. Both analysis methods were run with default parameters. For SIFT, we consider an amino acid substitution to be damaging when the score is <= 0.05, and tolerated if the score is > 0.05. For PolyPhen2, we used the following thresholds for pathogenic classification: 5% / 10% false positive rate (FPR) for the HumDiv model and 10% / 20% FPR for HumVarmodel. Mutations with their posterior probability scores associated with estimated false positive rates at or below the first lower FPR value were predicted to be probably damaging. Mutations with the posterior probabilities associated with false positive rates at or below the second (higher) FPR value were predicted to be possibly damaging. Mutations with estimated false positive rates above the second (higher) FPR value were classified as benign. We identified the subset of deleterious mutations that were identified with either method.

We utilized several approaches to determine the mutations, somatic CNVs and cancer rearrangements affected known or putative cancer driver genes. First, our gene list with pathogenic cancer mutations was compared to the Cancer Census gene list and the Catalogue of Somatic Mutations in Cancer (COSMIC), cancer-associated gene lists curated as part of the Cancer Genome Project at the Sanger Institute [23]. Second, we researched the function of every cancer mutation-associated gene using NCBI Gene (http://www.ncbi.nlm.nih.gov/gene) and PubMed searches. Each search involved using "(gene name) cancer" as the search term. Third, we conducted a gene ontology (GO) term analysis using g:profiler (http://biit.cs.ut.ee/gprofiler/) on the genes with high confidence cancer mutation calls [72]. Finally, we used KEGG PATHWAY mapping (http://www.genome.jp/kegg/pathway.html) to identify pathways affected by our cancer mutations [73].

**Allelic analysis of mutations in the gastric cancer genomes**

To estimate the clonal derivation between the two cancer genomes, we used an allelic frequency analysis for mutations found either in the primary versus metastatic tumor. We considered all mutations with potential biological impact for the primary and metastatic tumors **Additional file 1, Table S5)**. Total read depths for the targeted resequencing validation are also listed. Based on the method of Ding et al. [74], this analysis involves determining the proportion of reads containing the cancer mutation-of-interest among the different cancer genomes in comparison to the normal tissue. First, based on pathologic examination, we estimated the tumor sample cellularity given the presence of normal stroma. From the aligned BAM files, we extracted and counted the sequence reads containing either the mutation or wildtype allele for any given position. For each tumor genome, we calculated an allelic ratio for each mutation using the counts of the mutation allele reads versus the wildtype allele reads, adjusting for the tumor composition. From this analysis, we report the minor allelic frequency of each mutation for the primary and metastatic tumor. This analysis was conducted on the whole genome, exome and targeted resequencing data sets for the normal, primary tumor and metastatic tumor. Among all three sequencing approaches and their data sets, our results regarding fractional composition were concordant. We generally used the targeted resequencing data set and we also report the total read depth for each cancer somatic variant position that was evaluated **(Additional file 1; Table S5)**.

**SNP array analysis**

Standard protocols for DNA preparation, array hybridization and scanning were used to analyze the normal, primary tumor and metastatic tumor samples using SNP 6.0 arrays (Affymetrix, Santa Clara, CA). We used 1 µg of genomic DNA from our samples for array hybridization. Data analysis was performed using the Genotyping Console software and Birdseed V2 algorithm. We used 30 additional microarray data sets in concert with the studied samples to assess the quality of the SNP calls. SNP 6.0 array data was filtered using P-value threshold of

0.01. We used the Matlab programming language (Mathworks) for quantile probe normalization, probe level summarization and estimation of the copy number between a tumor and normal samples using log*2* ratio of the normalized signals (http://www.mathworks.com/products/bioinfo/examples.html?file=/products/demos/shipping/bioinfo/affysnpcnvdemo.html). Normal DNA from peripheral leukocytes and normal gastric tissue was used for SNP analysis and compared to insure concordance.

**Table S1.** Summary of normal, tumor, and metastasis exome and whole genome sequencing coverage.

| Genome sequencing approach | Exome | | | Whole Genome | | |
|---|---|---|---|---|---|---|
| **Samples** | **Normal** | **Primary Tumor** | **Metastatic Tumor** | **Normal** | **Primary Tumor** | **Metastatic Tumor** |
| **Mapped sequence (Gb)** | 5.1 | 4.6 | 4.5 | 145.2 | 253.7 | 93 |
| **Average haploid coverage** | 141.4X | 126.6X | 124.5X | 46.8X | 81.8X | 30.0X |
| **Coverage of exome or genome (%)** | 99.1% | 99.1% | 99.1% | 91.6% | 91.6% | 91.6% |
| **10X or greater sequence coverage (%)** | 96.6% | 96.6% | 96.8% | 91.1% | 91.3% | 90.3% |
| **30X or greater sequence coverage (%)** | 90.9% | 89.8% | 90.9% | 86.6% | 90.4% | 51.7% |
| **60X or greater sequence coverage (%)** | 78.6% | 74.8% | 77.0% | 21.8% | 82.4% | 2.1% |

**Table S2.** Single nucleotide variant (SNV) and small indel calling from genome sequencing data sets.

| Genome Sequencing Approach | Exome | | | Whole Genome | | |
|---|---|---|---|---|---|---|
| Sample | Normal | Primary Tumor | Metastasis | Normal | Primary Tumor | Metastasis |
| Combined Homozygote and Heterozygote SNVs and indels | 22,393 | 22,430 | 22,453 | 4,322,887 | 4,309,825 | 4,303,636 |
| Called somatic mutations in coding or regulatory regions | NA | 49 | 49 | NA | 111 | See exome data |
| Validated somatic mutations in coding or regulatory regions * | NA | 30 | 43 | NA | 31 | See exome data |

***Excluding the *CDH1* deletion**

**Table S3.** Summary of the validated cancer copy number variations and loss-of-heterozygosity (LOH) events.

| Origin | Chr | Chromosome interval Positions | | Amplification interval genes | Copy number aberration | Copy number | LOH |
|---|---|---|---|---|---|---|---|
| Unique to the Primary Tumor | 5 | 113893866 | 116091064 | *TRIM36, PGGT1B, CCDC112, FEM1C, TICAM2, TMED7, CDO1, ATG12, AP3S1, AQPEP, COMMD10, SEM6A* | Amplification | 3 | |
| | 10 | 121746196 | 123406274 | *PPAPDC1A, WDR11, FGFR2* | Amplification | 6 | |
| | 17 | 0 | 25308760 | | Deletion | 1 | Yes |
| Unique to the Metastasis | 2 | 87622839 | 87730893 | | Amplification | 5 | |
| | 3 | 0 | 60602587 | | Deletion | 1 | Yes |
| | 3 | 65344576 | 83311995 | | Deletion | 1 | Yes |
| | 4 | 0 | 165961802 | | Copy Neutral | 2 | Yes |
| | 4 | 170050291 | 175180921 | | Copy Neutral | 2 | Yes |
| | 5 | 163743617 | 180915260 | | Copy Neutral | 2 | Yes |
| | 11 | 2356587 | 4842345 | | Deletion | 1 | Yes |
| | 11 | 18369548 | 19042392 | | Deletion | 1 | Yes |
| | 14 | Entire Chromosome | | | Copy Neutral | 2 | Yes |
| | 16 | 78477033 | 90354753 | | Deletion | 1 | Yes |
| | 17 | 25308760 | 81061855 | | Deletion | 1 | Yes |
| | 18 | 0 | 33740982 | | Deletion | 1 | Yes |
| | 19 | 53059126 | 53432544 | | Deletion | 1 | Yes |
| | 20 | Entire Chromosome | | | Copy Neutral | 2 | Yes |
| | 22 | Entire Chromosome | | | Copy Neutral | 2 | Yes |

**Table S4.** Summary of the cancer rearrangement events.

| Origin | Structural Variation | Chr | Chromosome Interval Positions | |
|---|---|---|---|---|
| Common to the Primary and Metastatic Tumor | Deletion | 7 | 38386883 | 38397600 |
| | Deletion | 15 | 100686976 | 100693180 |
| | Deletion | 19 | 21268385 | 21334585 |

| | | | | |
|---|---|---|---|---|
| Unique to the Primary Tumor | Deletion | 5 | 42628385 | 42631158 |
| | Inversion | 15 | 99301834 | 101056862 |
| | Inversion | 16 | 55794659 | 55867118 |
| | Deletion | 19 | 6493052 | 6498221 |
| | Deletion | 22 | 23959245 | 23965974 |

## Table S5. Validated cancer mutations and analysis of mutant allelic frequency.

| Origin | Gene | Chr | Position | Mutation | Amino acid alteration | Mutation class | Predicted impact on gene product | Normal tissue mutation allelic fraction | Primary tumor mutation allelic fraction | Metastasis mutation allelic fraction | Normal tissue total sequence depth at mutation | Primary tumor total sequence depth at mutation | Metastasis total sequence depth at mutation |
|---|---|---|---|---|---|---|---|---|---|---|---|---|---|
| **Common to the Primary Tumor and Metastasis** | *LZIC* | 1 | 9995482 | T>A | NA | intron | | 0.00 | 0.26 | 0.18 | 110 | 105 | 47 |
| | *OVGP1* | 1 | 111959018 | G>A | D425D | coding-synonymous | | 0.00 | 0.43 | 0.59 | 364 | 324 | 235 |
| | *SETDB1* | 1 | 150917623 | +G | frameshift | insertion | DAMAGING | 0.00 | 0.17 | 0.42 | 407 | 269 | 529 |
| | *LINGO4* | 1 | 151773767 | G>A | R472C | missense | DAMAGING | 0.00 | 0.51 | 0.53 | 175 | 153 | 82 |
| | *DSTYK* | 1 | 205130347 | A>T | NA | intron | | 0.00 | 0.32 | 0.13 | 128 | 126 | 76 |
| | *NR4A2* | 2 | 157186267 | G>A | D144D | coding-synonymous | | 0.00 | 0.28 | 1.00 | 86 | 118 | 20 |
| | *TTN* | 2 | 179422800 | T>A | E20221V | missense | DAMAGING | 0.00 | 0.36 | 0.66 | 378 | 387 | 179 |
| | *CASP10* | 2 | 202082269 | G>C | NA | intron | | 0.00 | 0.41 | 0.56 | 78 | 55 | 27 |
| | *ATG16L1* | 2 | 234182297 | G>T | NA | intron | | 0.00 | 0.35 | 0.37 | 475 | 444 | 282 |
| | *CCDC80* | 3 | 112358376 | C>T | R126Q | missense | | 0.00 | 0.27 | 0.51 | 322 | 280 | 179 |
| | *CAMK2A* | 5 | 149618240 | C>T | NA | intron | | 0.00 | 0.51 | 0.30 | 330 | 300 | 128 |
| | *FILIP1* | 6 | 76072536 | C>T | R125Q | missense | | 0.00 | 0.42 | 0.34 | 295 | 318 | 194 |
| | *MYO6* | 6 | 76551096 | G>A | NA | splice-5 | DAMAGING | 0.00 | 0.23 | 0.20 | 79 | 65 | 41 |
| | *LPL* | 8 | 19805707 | C>T | NA | coding-synonymous | | 0.00 | 0.31 | 0.50 | 682 | 621 | 432 |
| | *DOCK5* | 8 | 25154053 | G>A | V165V | coding-synonymous | | 0.00 | 0.57 | 0.28 | 219 | 181 | 90 |
| | *MPDZ* | 9 | 13219560 | G>A | R362W | missense | DAMAGING | 0.00 | 0.40 | 0.41 | 238 | 187 | 109 |
| | *TNC* | 9 | 117853243 | C>T | A19T | missense | | 0.00 | 0.27 | 0.22 | 316 | 296 | 152 |
| | *OR5M11* | 11 | 56310065 | G>T | A223A | coding-synonymous | | 0.00 | 0.48 | 0.57 | 215 | 177 | 85 |
| | *VWCE* | 11 | 61045928 | C>T | V449I | missense | | 0.00 | 0.40 | 0.50 | 401 | 337 | 209 |
| | *B4GALNT3* | 12 | 662873 | C>T | A595V | missense | | 0.00 | 0.39 | 0.53 | 153 | 154 | 78 |
| | *CACNA2D4* | 12 | 1904446 | G>A | NA | intron | | 0.00 | 0.46 | 0.45 | 311 | 304 | 137 |
| | *ITPR2* | 12 | 26868250 | T>A | K279N | missense | | 0.00 | 0.46 | 0.43 | 531 | 561 | 282 |
| | *SCAPER* | 15 | 77064060 | C>G | NA | intron | | 0.00 | 0.29 | 0.35 | 377 | 315 | 256 |
| | *RRAD* | 16 | 66955948 | C>T | NA | utr-3 | | 0.00 | 0.60 | 0.19 | 273 | 230 | 108 |
| | *CDH1* | 16 | 68847326 - 68847403 | Deletion | NA | deletion | DAMAGING | 0.00 | 0.50 | 0.50 | 56 | 34 | 40 |

**Table S5. Validated cancer mutations and analysis of mutant allelic frequency.**

| Origin | Gene | Chr | Position | Mutation | Amino acid alteration | Mutation class | Predicted impact on gene product | Normal tissue mutation allelic fraction | Primary tumor mutation allelic fraction | Metastasis mutation allelic fraction | Normal tissue total sequence depth at mutation | Primary tumor total sequence depth at mutation | Metastasis total sequence depth at mutation |
|---|---|---|---|---|---|---|---|---|---|---|---|---|---|
| Common to the Primary Tumor and Metastasis | *TP53* | 17 | 7578370 | C>T | NA | splice-5 | DAMAGING | 0.00 | 0.64 | 0.67 | 563 | 464 | 178 |
| | *HAPLN4* | 19 | 19369385 | C>T | R255H | missense | DAMAGING | 0.00 | 0.12 | 0.78 | 49 | 66 | 23 |
| | *TGM3* | 20 | 2321085 | C>T | P647L | missense | | 0.00 | 0.43 | 0.87 | 156 | 163 | 100 |
| | *FERMT1* | 20 | 6096581 | G>A | L88F | missense | DAMAGING | 0.00 | 0.25 | 0.28 | 235 | 275 | 108 |
| | *BMP7* | 20 | 55758840 | C>T | R299H | missense | DAMAGING | 0.00 | 0.55 | 0.95 | 170 | 182 | 80 |
| | *NLGN3* | 23 | 70387298 | C>T | R451* | nonsense | DAMAGING | 0.00 | 0.11 | 0.54 | 377 | 384 | 189 |
| | *TAF1* | 23 | 70626561 | C>T | R1378* | nonsense | DAMAGING | 0.00 | 0.51 | 0.73 | 351 | 301 | 64 |

| | | | | | | | | | | | | | |
|---|---|---|---|---|---|---|---|---|---|---|---|---|---|
| Unique to the Primary Tumor | *ROBO2* | 3 | 77147398 | C>T | R99C | missense | DAMAGING | 0.00 | 0.35 | 0.00 | 270 | 193 | 95 |
| | *TMEM165* | 4 | 56277957 | C>T | T128T | coding-synonymous | | 0.00 | 0.29 | 0.00 | 297 | 288 | 105 |
| | *PRSS12* | 4 | 119256779 | C>T | P223P | coding-synonymous | | 0.00 | 0.16 | 0.00 | 558 | 375 | 244 |
| | *DYNC1H1* | 14 | 102474581 | C>T | R1962C | missense | DAMAGING | 0.00 | 0.19 | 0.00 | 135 | 135 | 40 |
| | *SEZ6L2* | 16 | 29896873 | C>T | NA | intron | | 0.00 | 0.17 | 0.00 | 193 | 219 | 96 |
| | *ZNF423* | 16 | 49557509 | G>A | NA | intron | | 0.00 | 0.40 | 0.00 | 192 | 241 | 123 |
| | *ATP2C2* | 16 | 84492748 | G>C | G724R | missense | DAMAGING | 0.00 | 0.41 | 0.00 | 201 | 164 | 79 |
| | *CECR2* | 22 | 18028506 | C>T | R1156C | missense | DAMAGING | 0.00 | 0.49 | 0.00 | 239 | 253 | 88 |

| | | | | | | | | | | | | | |
|---|---|---|---|---|---|---|---|---|---|---|---|---|---|
| Unique or expanded in the Metastasis | *NPHP4* | 1 | 5935112 | C>T | A956T | missense | | 0.00 | 0.00 | 0.18 | 61 | 97 | 37 |
| | *SPTA1* | 1 | 158623188 | G>A | H1022Y | missense | | 0.00 | 0.05 | 0.68 | 137 | 111 | 79 |
| | *LCT* | 2 | 136569954 | C>T | G760G | coding-synonymous | | 0.00 | 0.00 | 0.24 | 390 | 329 | 237 |
| | *PDE1A* | 2 | 183387006 | C>A | G33V | missense | | 0.00 | 0.02 | 0.38 | 308 | 329 | 195 |
| | *COL4A3* | 2 | 228159777 | G>A | T1172T | coding-synonymous | | 0.00 | 0.00 | 0.30 | 328 | 340 | 146 |
| | *TGFBR2* | 3 | 30691871 | -AA | frameshift | deletion | DAMAGING | 0.00 | 0.00 | 1.00 | 888 | 461 | 1237 |
| | *C3orf77* | 3 | 44283826 | C>T | S94L | missense | | 0.00 | 0.00 | 0.54 | 82 | 64 | 46 |
| | *SORCS2* | 4 | 7691291 | G>A | A351T | missense | DAMAGING | 0.00 | 0.04 | 0.66 | 242 | 192 | 78 |

## Table S5. Validated cancer mutations and analysis of mutant allelic frequency.

| Origin | Gene | Chr | Position | Mutation | Amino acid alteration | Mutation class | Predicted impact on gene product | Normal tissue mutation allelic fraction | Primary tumor mutation allelic fraction | Metastasis mutation allelic fraction | Normal tissue total sequence depth at mutation | Primary tumor total sequence depth at mutation | Metastasis total sequence depth at mutation |
|---|---|---|---|---|---|---|---|---|---|---|---|---|---|
| Unique or expanded in the Metastasis | *PCDH7* | 4 | 30723305 | C>G | S87R | missense | DAMAGING | 0.00 | 0.00 | 0.77 | 36 | 20 | 13 |
| | *GRID2* | 4 | 94031969 | A>T | V200V | coding-synonymous | | 0.00 | 0.07 | 0.59 | 230 | 253 | 107 |
| | *KIAA1109* | 4 | 123168497 | G>A | G1833S | missense | | 0.00 | 0.00 | 0.23 | 267 | 233 | 94 |
| | *FAT4* | 4 | 126238952 | G>A | V462V | coding-synonymous | | 0.00 | 0.05 | 0.32 | 126 | 99 | 36 |
| | *CDH12* | 5 | 21760747 | G>A | S518L | missense | DAMAGING | 0.00 | 0.08 | 0.42 | 176 | 196 | 116 |
| | *FBN2* | 5 | 127627326 | C>G | D2063H | missense | DAMAGING | 0.00 | 0.02 | 0.33 | 416 | 304 | 210 |
| | *CAP2* | 6 | 17507405 | C>T | D102D | coding-synonymous | | 0.00 | 0.00 | 0.11 | 453 | 406 | 253 |
| | *COL12A1* | 6 | 75843032 | C>T | R1924H | missense | DAMAGING | 0.00 | 0.00 | 0.19 | 235 | 202 | 133 |
| | *SLC22A2* | 6 | 160679409 | C>T | V106V | coding-synonymous | | 0.00 | 0.00 | 0.29 | 93 | 132 | 62 |
| | *INTS10* | 8 | 19690805 | G>A | T501T | coding-synonymous | | 0.00 | 0.00 | 0.29 | 165 | 173 | 87 |
| | *UBAP2* | 9 | 33923045 | C>T | | intron | | 0.00 | 0.00 | 0.27 | 197 | 203 | 68 |
| | *SLC28A3* | 9 | 86909179 | G>A | I291I | coding-synonymous | | 0.00 | 0.06 | 0.14 | 384 | 332 | 146 |
| | *ABL1* | 9 | 133755519 | -T | frameshift | deletion | DAMAGING | 0.00 | 0.00 | 0.72 | 426 | 149 | 543 |
| | *DOCK1* | 10 | 128795088 | C>T | L184F | missense | DAMAGING | 0.00 | 0.00 | 0.17 | 319 | 271 | 162 |
| | *FAM118B* | 11 | 126120571 | G>A | L170L | coding-synonymous | | 0.00 | 0.00 | 0.40 | 407 | 286 | 181 |
| | *PZP* | 12 | 9307409 | G>A | Q1193* | nonsense | DAMAGING | 0.00 | 0.00 | 0.16 | 123 | 134 | 106 |
| | *ABCC9* | 12 | 22086799 | C>T | P67P | coding-synonymous | | 0.00 | 0.00 | 0.22 | 184 | 216 | 98 |
| | *LRRC43* | 12 | 122670797 | G>A | A158T | missense | | 0.00 | 0.04 | 0.30 | 243 | 203 | 62 |
| | *TMEM132D* | 12 | 129558541 | G>A | P1060L | missense | DAMAGING | 0.00 | 0.00 | 0.33 | 576 | 556 | 356 |
| | *NBEA* | 13 | 36046612 | C>T | T2175M | missense | DAMAGING | 0.00 | 0.00 | 0.40 | 440 | 343 | 120 |
| | *GABRA5* | 15 | 27182365 | G>A | W205* | nonsense | DAMAGING | 0.00 | 0.01 | 0.43 | 205 | 187 | 81 |
| | *PLA2G4E* | 15 | 42298192 | G>A | P174L | missense | DAMAGING | 0.00 | 0.00 | 0.40 | 219 | 214 | 99 |
| | *ADAL* | 15 | 43641114 | +A | frameshift | insertion | DAMAGING | 0.00 | 0.00 | 0.24 | 245 | 180 | 98 |
| | *MMP15* | 16 | 58079179 | G>T | V613V | coding-synonymous | | 0.01 | 0.04 | 0.32 | 93 | 131 | 41 |
| | *DNAH9* | 17 | 11797798 | G>A | A3797A | coding-synonymous | | 0.00 | 0.00 | 0.30 | 555 | 443 | 175 |

**Table S5. Validated cancer mutations and analysis of mutant allelic frequency.**

| Origin | Gene | Chr | Position | Mutation | Amino acid alteration | Mutation class | Predicted impact on gene product | Normal tissue mutation allelic fraction | Primary tumor mutation allelic fraction | Metastasis mutation allelic fraction | Normal tissue total sequence depth at mutation | Primary tumor total sequence depth at mutation | Metastasis total sequence depth at mutation |
|---|---|---|---|---|---|---|---|---|---|---|---|---|---|
| Unique or expanded in the Metastasis | *CCDC46* | 17 | 63957650 | G>A | A601A | coding-synonymous | | 0.00 | 0.01 | 0.59 | 403 | 389 | 170 |
| | *TBC1D17* | 19 | 50387814 | G>A | V448M | missense | DAMAGING | 0.00 | 0.00 | 0.34 | 258 | 324 | 166 |
| | *DCX* | 23 | 110644391 | G>A | R259C | missense | DAMAGING | 0.00 | 0.03 | 0.56 | 251 | 284 | 66 |
| | *GPC4* | 23 | 132458559 | A>G | F109L | missense | DAMAGING | 0.00 | 0.00 | 0.67 | 170 | 197 | 85 |

**Table S6.** Microsatelllite genetic markers assessed for microsatellite instability.

| Gene or marker | Genomic location | Microsatellite sequence |
|---|---|---|
| **Exon-based microsatellite** | | |
| *TGFBR2* | chr3:30691868-30691885 | AAGGAAAAAAAAAAGCCT |
| *ACVR2* | chr2:148683682-148683697 | GCATAAAAAAAAGAGG |
| *MSH3* | chr5:79970911-79970926 | GGACAAAAAAAAGGGC |
| *MYH11* | chr16:15802683-15802698 | CTGTGGGGGGGGCCCT |
| *SEC31A* | chr4:83785561-83785577 | TCAATTTTTTTTTGGCA |
| *CLOCK* | chr4:56336950-56336966 | TGCTAAAAAAAAACCAT |
| *GLYR1* | chr16:4862225-4862240 | AGCGCCCCCCCCTGGA |
| *ABCC5* | chr3:183665253-183665269 | AAACAAAAAAAAAGGAG |
| *WDTC1* | chr1:27621104-27621119 | ACATGGGGGGGGAACA |
| *UBR5* | chr8:103289345-103289360 | CTTCTTTTTTTTGCCG |
| *PRDM2* | chr1:14108745-14108761 | TCCCAAAAAAAAAGTTT |
| *WASF3* | chr13:27255383-27255398 | GCCGCCCCCCCCGCCT |
| *ROCK1* | chr18:18533675-18533690 | CCCATTTTTTTTGTTC |
| *OR51E2* | chr11:4703471-4703486 | TGGGAAAAAAAAGAGG |
| *HPS1* | chr10:100186983-100186998 | CCATGGGGGGGGTGCC |
| *TCEB3* | chr1:24078399-24078415 | ACTTAAAAAAAAATGAC |
| *LARP7* | chr4:113570750-113570765 | CTTTAAAAAAAACAAT |
| *PIGB* | chr15:55631499-55631514 | CGTATTTTTTTTGGCC |
| *ZBTB20* | chr3:114057999-114058013 | TGCAGGGGGGGTCCC |
| *AKAP9* | chr7:91603081-91603096 | GCAGAAAAAAAAGAGA |

| Gene or marker | Genomic location | Microsatellite sequence |
|---|---|---|
| **Non-coding microsatellite** | | |
| BAT25 | chr2:47642304-47642353 | AAGGTTTTTTTTTTTTTTTTTTTTTT<br>TGAGA |
| BAT26 | chr4:55598200-55598249 | TTGATTTTTTTTTTTTTTTTTTTTTTT<br>TTGAGA |
| D5S346 | chr5:112213624-112213748 | ACTCTGTGTGTGTGTGTGTGTGTG<br>TGTGTGTGTGTGTGTGTGTGTAAA<br>T |
| D2S123 | chr2:51288378-51288657 | TGATACACACACACACACACACAC<br>ACACACACACACACACACACACAT<br>ATT |
| D17S250 | chr17:37152091-37152341 | GAAAGTGTGTGTGTGTGTGTGTGT<br>GTGTGTGTGTGTGTGTGTGTTTGA |

**Table S7.** Gastric cancer cell line IC50s for different gastric cancer cell lines. The IC50s of cytotoxic chemotherapies (paclitaxel, carboplatin, 5-fluorouracil) or an FGFR2 small molecule tyrosine kinase inhibitor (AZD4547) are listed.

| **Drug** | **Kato III IC50** | **AGS IC50** |
|---|---|---|
| **AZD4547** | 2nM | 39,580nM |
| **Paclitaxel** | 78nM | 53nM |
| **Carboplatinum** | 1.6uM | 3.2uM |
| **5-fluorouracil** | 743nM | 163nM |

Figure S1. Nadauld, et al.

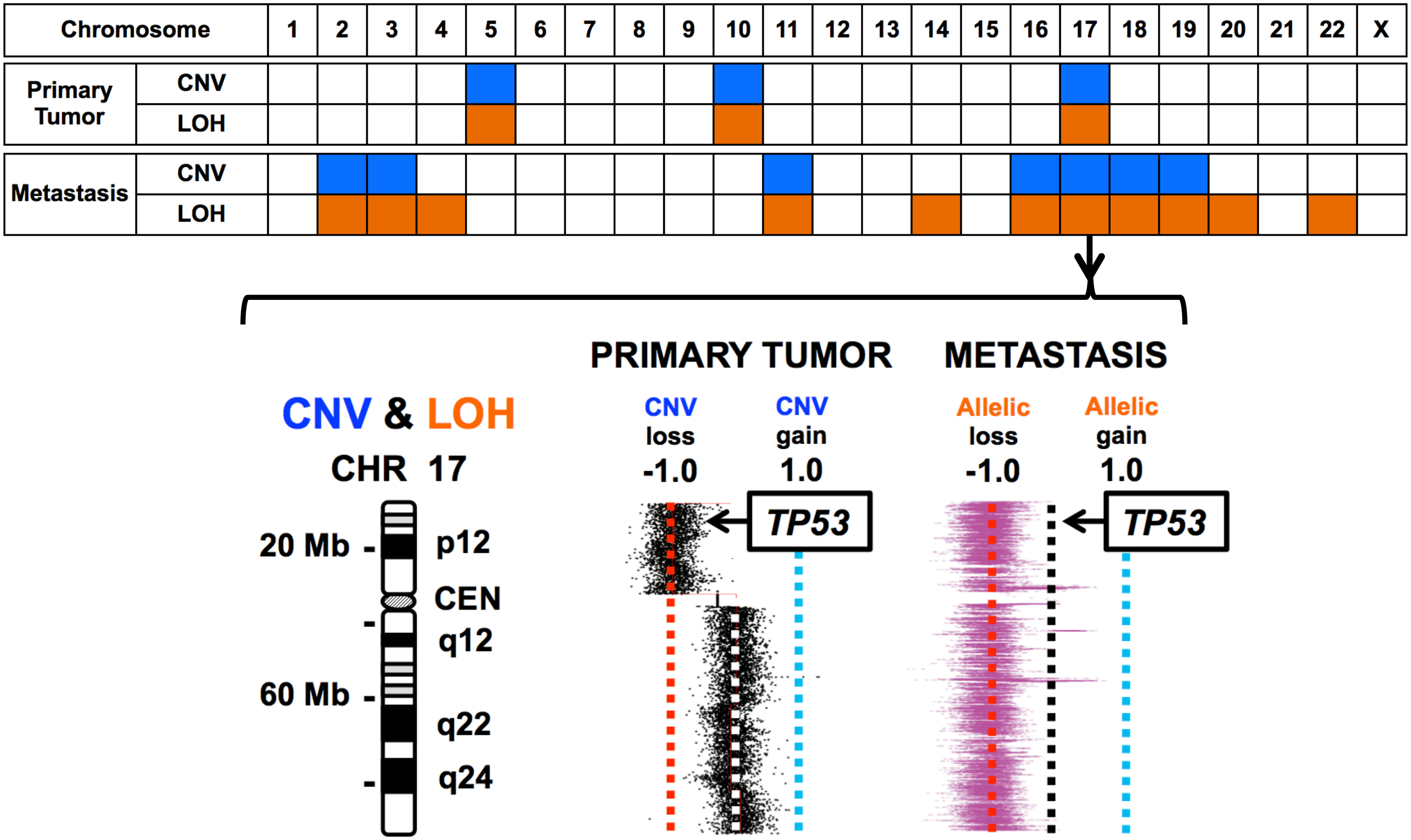


**Figure S1. Biallelic events in the *TP53* loci and gene.** For Chromosome 17, copy number variation (CNV) and loss-of-heterozygosity (LOH) is mapped with a cancer driver mutation in *TP53*. For CNVs, the X axis indicate the CNVs of the cancer to matched normal genome iusing a normalized read depth. The Y axis designates position on the respective chromosome which is shown to the left of the copy number profile. LOH is indicated by a SNP minor allele fraction ratio over the metastasis over the normal.Mutations are shown as boxed arrows with the gene symbol. Both the primary tumor and metastasis had biallelic events involving a common *TP53* point mutation and deletions. In the case of the primary tumor a genomic deletion occurred in the p arm as shown by a CNV value of -1 which indicates hemizygous loss. In the metastasis, this deletion was extended to involve most of the q arm as seen by extensive LOH with a value of -1 when comparing the metastasis to the normal genome SNPs.

Figure S2. Nadauld, et al.

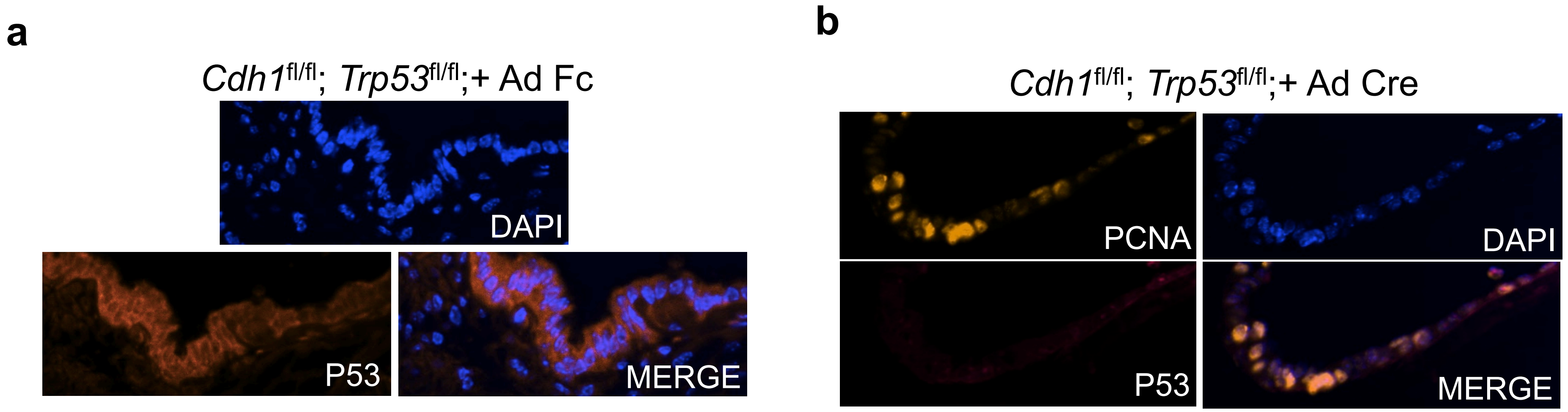


**Figure S2. Deletion of CDH1 and TRP53 expression in gastric organoids.**

(a) *Cdh1*$^{fl/fl}$; *Trp53*$^{fl/fl}$ gastric organoid cultures were infected with Fc-expressing adenovirus and imaged by immunofluorescence with antibodies against CDH1 and TRP53.

(b) *Cdh1*$^{fl/fl}$; *Trp53*$^{fl/fl}$ gastric organoid cultures were infected with CreGFP-expressing adenovirus and imaged by immunofluorescence with antibodies against PCNA or TRP53.

Figure S3. Nadauld, et al.

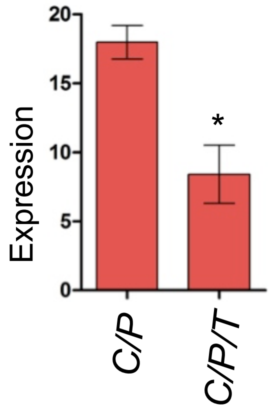


**Figure S3. shRNA-mediated knockdown of TGFBR2.**

RT-PCR for *Tgfbr2* using total RNA from CDH1/TRP53-deficient gastric organoids infected with GFP-expressing retrovirus (C/P), or with retrovirus expressing shRNA against CDH1/TRP53-deficient gastric organoids that also express *Tgfbr2 shRNA* (C/P/T). Expression is relative to total RNA and error bars indicate standard deviation. Asterisk indicates statistically significant difference, p=0.017.
